# From Coils to Crawls: A Snake-Inspired Soft Robot for Multimodal Locomotion and Grasping

**DOI:** 10.1007/s40820-025-01762-9

**Published:** 2025-04-30

**Authors:** He Chen, Zhong Chen, Zonglin Liu, Jinhua Xiong, Qian Yan, Teng Fei, Xu Zhao, Fuhua Xue, Haowen Zheng, Huanxin Lian, Yunxiang Chen, Liangliang Xu, Qingyu Peng, Xiaodong He

**Affiliations:** 1 National Key Laboratory of Science and Technology On Advanced Composites in Special Environments, Center for Composite Materials and Structures, Harbin Institute of Technology, Harbin, 150080 People’s Republic of China; 2 Dongfang Electric Academy of Science and Technology Co. Ltd, Chengdu, 611731 People’s Republic of China; 3 Suzhou Research Institute of HIT, Suzhou, 215104 People’s Republic of China

**Keywords:** Untethered biomimetic robots, Coiling deformation, Multimodal locomotion, Multistimuli-response, Coiling grasping

## Abstract

Currently, numerous biomimetic robots inspired by natural biological systems have been developed. However, creating soft robots with versatile locomotion modes remains a significant challenge. Snakes, as invertebrate reptiles, exhibit diverse and powerful locomotion abilities, including prey constriction, sidewinding, accordion locomotion, and winding climbing, making them a focus of robotics research. In this study, we present a snake-inspired soft robot with an initial coiling structure, fabricated using MXene-cellulose nanofiber ink printed on pre-expanded polyethylene film through direct ink writing technology. The controllable fabrication of initial coiling structure soft robot (ICSBot) has been achieved through theoretical calculations and finite element analysis to predict and analyze the initial structure of ICSBot, and programmable ICSBot has been designed and fabricated. This robot functions as a coiling gripper capable of grasping objects with complex shapes under near infrared light stimulation. Additionally, it demonstrates multi-modal crawling locomotion in various environments, including confined spaces, unstructured terrains, and both inside and outside tubes. These results offer a novel strategy for designing and fabricating coiling-structured soft robots and highlight their potential applications in smart and multifunctional robotics.

**Supplementary Information:**

The online version contains supplementary material available at 10.1007/s40820-025-01762-9.

## Highlights

- A tunable initial coiling structure soft robot (ICSBot) has been developed by combining theoretical calculations, finite element analysis, and direct ink writing technology.
- By mimicking the prey-handling behavior of snakes, ICSBot functions as a coiling gripper, capable of dynamic uncoiling, object grasping, and controlled release.
- By simulating the multimodal movement of snakes, ICSBot demonstrated sidewinding locomotion to navigate unstructured environments, accordion locomotion to navigate narrow tubes, and winding climbing locomotion to traverse tubular structures under near-infrared light radiation.

**Supplementary Information:**

The online version contains supplementary material available at 10.1007/s40820-025-01762-9.

## Graphical abstract

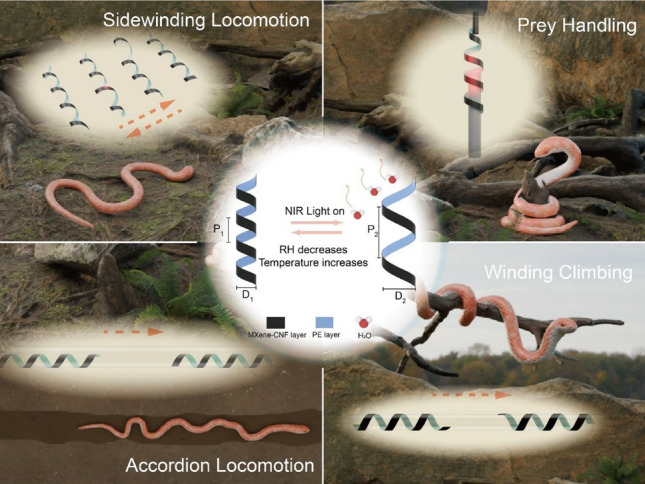

## Introduction

Soft robots have drawn extensive inspiration from biological systems in nature, leveraging the diverse locomotion modes of various organisms [1, 2]. By mimicking these biological movements, numerous untethered soft robots have been developed to perform tasks in different environments, including insect larva-inspired jumping robots [3], monkey-inspired climbing robots [4], centipede-inspired multi-sensing crawling robots [5], and inchworm-inspired crawling robots [6]. Despite these advancements, existing biomimetic soft robots face significant limitations, such as restricted mobility, simple application scenarios, and reliance on single locomotion modes. This emphasizes the necessity of exploring biomimetic soft robots with enhanced mobility and broader applicability.

**Fig. 1:**
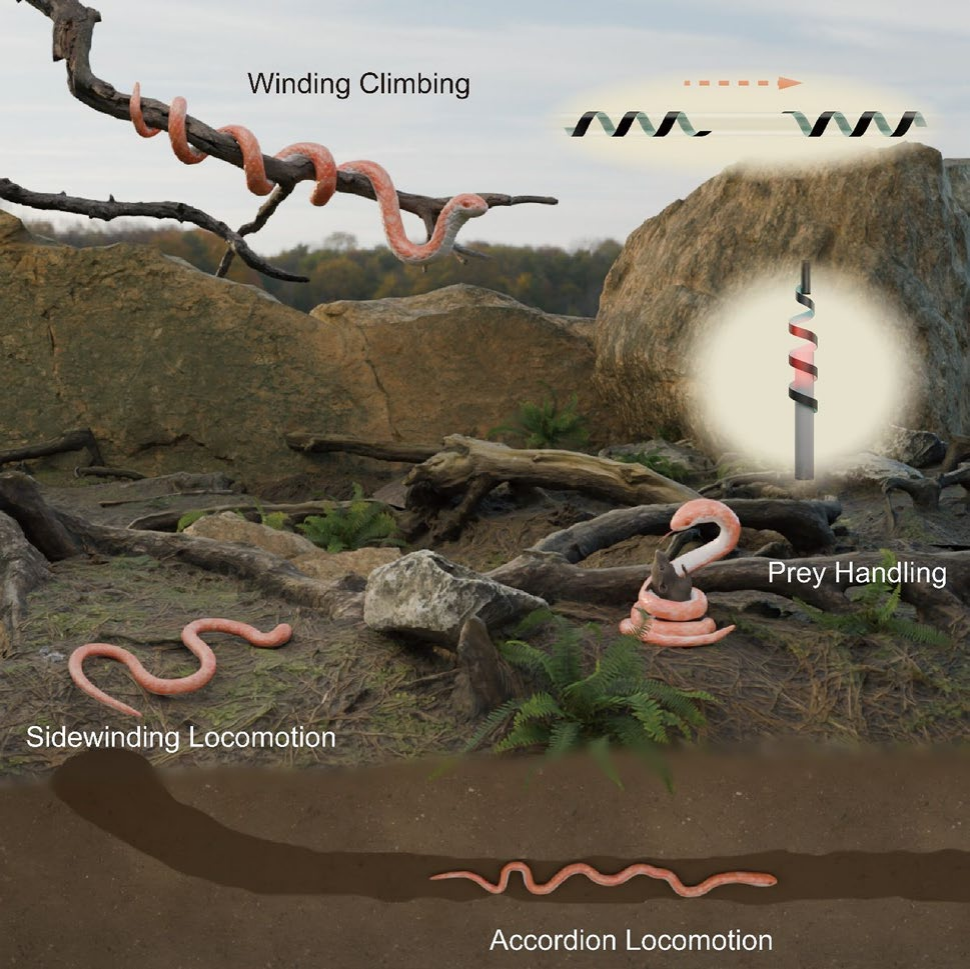
Snake-inspired ICSBot for coiling grasping and multimodal locomotion

Among biological systems, snakes represent a particularly compelling source of inspiration due to their evolutionarily refined multimodal locomotion. Snakes demonstrate a range of adaptive movement strategies depending on environmental conditions [7, 8]. As shown in Fig. 1, the snake wraps itself around its prey, continuously applying pressure until the prey loses its ability to move. In addition, snakes can move quickly on the ground through sidewinding locomotion. This locomotion greatly increases the friction between the abdomen of the snake and the ground, ensuring smooth locomotion of the snake in unstructured environments. When snakes are in narrow caves, they move through accordion locomotion, which is achieved by first anchoring the posterior body of the snake to the substrate, then extending the body until a new anchoring point is established in the anterior body, and finally contracting the body to move towards the anterior body. In arboreal settings, winding climbing locomotion allows snakes to ascend by alternately wrapping and unwrapping their body around branches. These unique multimodal locomotion strategies and prey-handling behaviors provide valuable insights for designing soft robots capable of navigating complex, unstructured environments.

In recent years, biomimetic robots with snake-like locomotion have been widely studied [9, 10]. For instance, inspired by the rectilinear locomotion of snakes, a soft robot with an origami skin structure was designed, which can crawl through directional friction caused by surface buckling [11]. Inspired by the winding climbing locomotion of snakes, a climbing robot consisting of two winding mechanisms and one telescopic mechanism was designed, which can quickly move on a vertical surface [12]. However, many existing snake-inspired robots are large and rely on pneumatic control, limiting their versatility and scalability. To address these challenges, the development of small-scale, untethered snake-like robots that achieve multimodal locomotion under non-contact stimulation is essential. For example, inspired by sidewinding locomotion, a soft robot with a coiling structure was developed, which can achieve autonomous sidewinding locomotion under heat stimulation [13]. Inspired by the accordion movement, a multi-layered soft robot was built that can perform accordion locomotion under light stimulation [14]. However, these soft robots can only imitate a single locomotion mode of snakes, lacking exploration of the ability to achieve multimodal locomotion of snake-like soft robots. To simulate the locomotion of snakes, it is feasible to design a soft robot with coiling deformation capability. Coiling deformation has a higher degree of freedom than 2D bending deformation, showing great application prospects in the field of soft robots, such as grasping [15–18], rolling [19, 20], self-sustained oscillation [21], aerial seedings [22], etc. Current methods for achieving coiling deformation primarily focus on material innovations, such as integrating photothermal conversion materials (e.g., carbon nanotube [23, 24], MXene [25], graphene [26]) with polyethylene (PE) films, or structural strategies that introduce alignment through patterned designs [27–29]. Among them, MXene materials have rich oxygen-containing functional groups, excellent photothermal conversion performance, and outstanding mechanical properties, which are widely used in the field of soft robots [30–32]. Nevertheless, many soft robots capable of coiling deformation have been developed; it remains a challenge to design and fabricate soft robots with controllable initial coiling structures and explore their application.

This study addresses these challenges by designing and fabricating a novel soft robot, termed ICSBot (initial coiling structure soft robot), inspired by snake locomotion and prey handling strategies. ICSBot is fabricated using direct ink writing (DIW) technology to print MXene-cellulose nanofiber (CNF) ink onto pre-expanded PE film. The initial coiling structure of ICSBot is predicted through theoretical calculations and finite element analysis (FEA), and verified through experiments. Leveraging the anisotropic thermal expansion properties of the PE layer and the photothermal conversion capability and water molecule adsorption/desorption properties of the MXene-CNF layer, ICSBot achieves reversible coiling deformation under stimuli such as near-infrared (NIR) light, humidity, or temperature changes. By mimicking the prey-handling behavior of snakes, ICSBot functions as a coiling gripper, capable of dynamic uncoiling, object grasping, and controlled release. Furthermore, ICSBot demonstrates sidewinding locomotion under periodic NIR irradiation, with direction and distance controllable via the NIR irradiation direction and printing angle. Additionally, ICSBot emulates accordion locomotion to navigate narrow tubes and winding climbing locomotion to traverse tubular structures, enabling it to operate effectively in complex, confined, and unstructured environments. This work presents a novel approach to designing soft robots with controllable initial coiling structures. Furthermore, the multimodal locomotion of ICSBot under non-contact stimulation (NIR light) greatly improves mobility and application scenarios compared to existing snake-like soft robots, providing new opportunities for applications in smart gripping and versatile crawling robotics.

## Experimental Section

The design and fabrication of three-dimensional (3D) initial structures for soft robots typically rely on three primary methods: the hot pressing setting process [26, 33, 34], the stimulation setting process [35, 36], and the solidification setting process [13, 37]. While these approaches have been widely adopted, they often involve multiple fabrication steps, resulting in increased complexity. To address these challenges, we developed a direct ink writing (DIW) technology capable of producing 3D initial structure soft robots in a simple and efficient manner [38]. Using this approach, we fabricated the initial coiling structure soft robot (ICSBot) by printing MXene-CNF ink onto pre-expanded polyethylene (PE) film. Leveraging the anisotropic deformation of the pre-expanded PE film, the resulting bilayer film exhibits a controllable initial coiling structure upon cooling to room temperature.

### Fabrication of Ti_3_C_2_T_***x***_ MXene

Ti_3_C_2_T_*x*_ MXene was fabricated using HCl/LiF mixed solution etching method. Specifically, 6.4 g of LiF (99%) was added to 80 mL of 9 M HCl and stirred thoroughly until completely dissolved. Ti_3_AlC_2_ powder (400 mesh) was slowly added to the HCl/LiF mixed solution and stirred continuously at 40 °C for 24 h. Then, multilayer Ti_3_C_2_T_*x*_ MXene precipitates were obtained by centrifuging the mixed solution multiple times until the pH value approached around 6. Multilayer Ti_3_C_2_T_*x*_ MXene was sonicated in an ice water bath under Ar atmosphere protection for 1 h. Then the Ti_3_C_2_T_*x*_ MXene dispersion was obtained by centrifugation at 3500 r min^−1^ for 1 h. Finally, Ti_3_C_2_T_*x*_ MXene dispersion was concentrated to an appropriate concentration for subsequent steps by centrifugation.

### Fabrication of MXene-CNF Ink

MXene and CNF were mixed in DI water in mass ratios of 9:1. The mass concentration of the mixed solution was made to be 3%–4% by adding DI water. Then MXene-CNF ink was fabricated by continuous stirring at room temperature for 4 h.

### Fabrication of ICSBot

The ICSBot was fabricated using DIW through a microelectronic printer (MP1100, Prtronic, Shanghai Mifang Electronic Technology Co., Ltd). Specifically, the PE film was placed on a pre-heated printing platform (T = 40, 50, and 60 °C) for 20 min to fully expand. MXene-CNF ink was added to a syringe with a needle. An air pump was then attached to the top of the syringe to extrude the ink. MXene-CNF ink was added to a syringe with a needle. The needle was moved to a position of 0.1 mm from the PE film. Then the MXene/CNF ink was extruded onto the pre-heated PE film (needle moving speed = 5 mm s^−1^) at different angles *θ* (*θ* = 15°, 30°, 40°, 60°, 75°) under a printing air pressure of 5–10 kPa. After the printed ink is dried, it is cut into long rectangular strips (80 mm × 2–8 mm) on the heated printing platform along with the printing angle. Then the bilayer film is quickly peeled off the platform, where the bilayer film undergoes a coiling deformation towards the PE layer. Finally, the coiling bilayer actuator is obtained.

### FEA Simulations

FEA was performed to simulate the initial morphology of the coiling bilayer actuator after different fabrication processes using the commercially available package Abaqus. Specifically, composite bilayer thin shell structures were used due to the ultra-thin geometry of the MXene-CNF layer and the PE layer. The deformation behavior induced by internal stresses during fabrication was modeled as that driven by the difference in thermal expansion of the bilayer. The constitutive model in the FEA simulations is assumed to be linear elastic. Orthogonal anisotropy is utilized to model the deformation produced by anisotropic coefficients of thermal expansion. The specific material properties used for the simulation are shown in Table S1. The MXene-CNF/PE bilayer structure was meshed using an eight-node doubly curved thin shell with reduced integration shell elements (S8R5).

### Characterization

Detail is provided in the supporting materials.

## Results and Discussion

### Design and Fabrication of ICSBot

PE film was selected as the substrate due to its high anisotropic coefficient of thermal expansion (CTE), a result of directional stretching during the blow molding process [26, 39]. MXene, known for its excellent photothermal conversion capability and water molecule adsorption/desorption properties, was synthesized using a modified HCl/LiF etching method (Note S1 and Fig. S1) [40–42]. To enhance mechanical performance and avoid MXene nanosheet restacking, CNF was incorporated into the ink [43]. The introduction of CNF increased the interlayer spacing of MXene flakes through hydrogen bonding, improving water molecule adsorption/desorption and mechanical stability (Note S2 and Figs. S2, S3). The resulting MXene-CNF ink demonstrated excellent rheological properties, ensuring compatibility with DIW technology (Note S3 and Fig. S4). MXene-CNF ink can be successfully printed on different polymer substrates (PLA, PET, and PI), demonstrating its adaptability for DIW and potential application in flexible devices (Fig. S5).

**Fig. 2:**
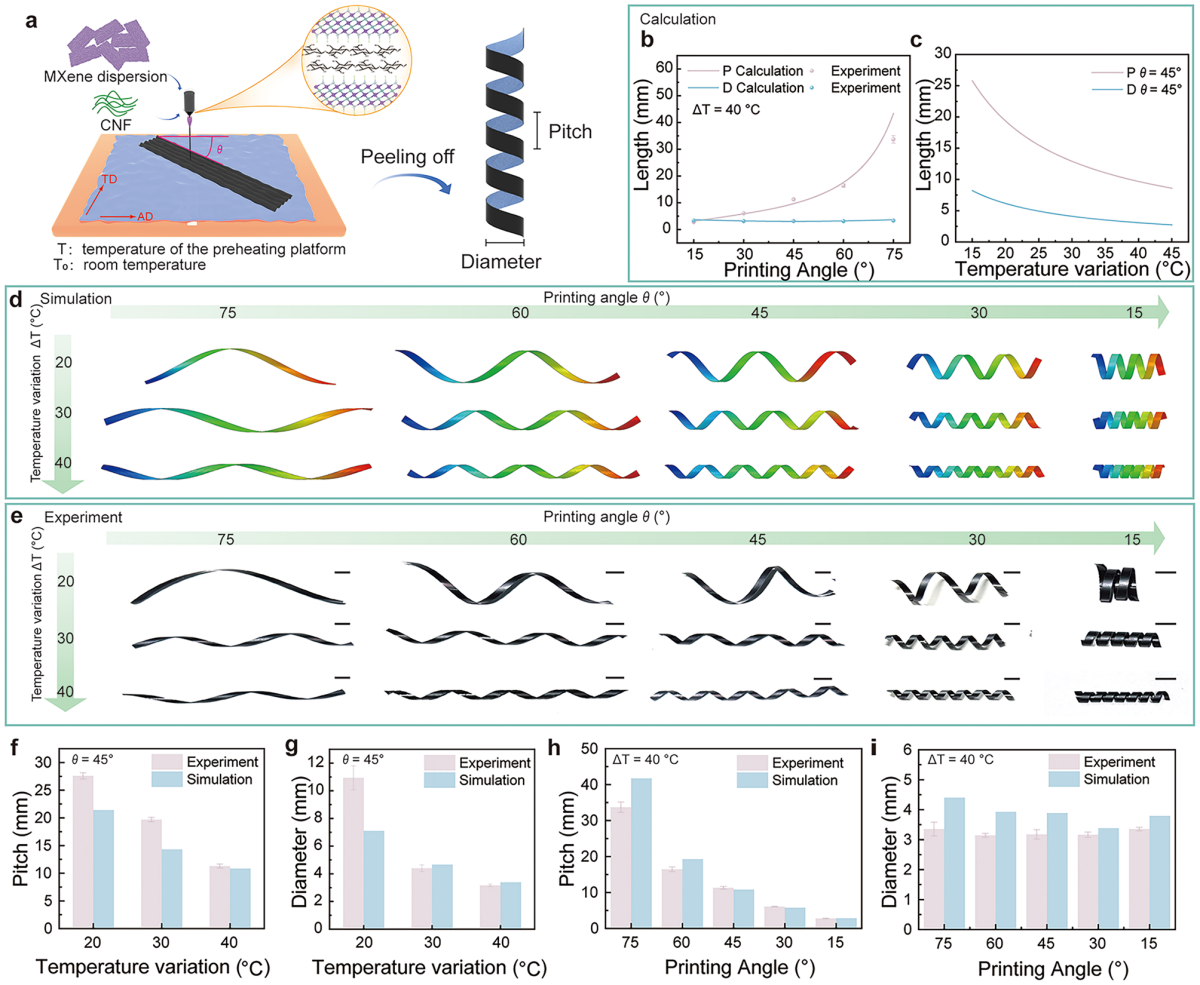
Design and fabrication of ICSBot. **a** Schematic illustration of the fabrication process of the ICSBot. **b**, **c** Dependence of pitch and diameter of ICSBot on (**b**) printing angle and (**c**) temperature variation under laminated composite material model. **d** FEA results of ICSBot under different printing angles and temperature variations. **e** Optical images of ICSBot under different printing angles and temperature variations. **f**, **g** Comparison between FEA and experimental results in (**f**) pitch and (**g**) diameter of ICSBot as a function of temperature variation (printing angle *θ* = 45°). **h**, **i** Comparison between FEA and experimental results in (**h**) pitch and (**i**) diameter of ICSBot as a function of printing angle (temperature variation ΔT = 40 °C). Scale bars, 5 mm

The fabrication process is schematically illustrated in Fig. 2a. Plasma-treated PE film was placed on a preheated printing platform, which enhanced its hydrophilicity and bonding compatibility with the ink (Fig. S6). MXene-CNF ink was then extruded through a syringe nozzle and printed onto the PE film in a rectangular pattern at a specific angle (*θ*) relative to the alignment direction (AD) of the PE film. Upon drying, the bilayer film was peeled off the platform, triggering a temperature decrease that caused the PE layer to shrink and the MXene-CNF layer to expand due to moisture absorption. This mismatch in volume changes induced coiling deformation, determined by the anisotropic CTE of the PE layer and the printing angle *θ*. The coiling deformation of ICSBot was further influenced by the temperature differential between the preheated platform (T) and room temperature (T_0_). The fabricated ICSBot exhibited a flexible structure with the MXene-CNF layer forming the outer surface and the PE layer forming the inner surface (Fig. S7). Cross-sectional SEM images confirmed tight bonding between the MXene-CNF and PE layers, showcasing the layered MXene structure (Figs. S8, S9). Surface SEM images revealed wrinkles that enhanced surface roughness and facilitated water molecule adsorption/desorption (Fig. S10). As the number of printed layers increased, the layers exhibited strong adhesion without gaps. The thickness of the MXene-CNF layer increased from 3.7 μm for a single layer to 7 and 11.3 μm for two and three layers, respectively (Fig. S11). The initial coiling structure of ICSBot can be controlled by printing parameters. As the printing speed increases, the pitch (P) and diameter (D) of ICSBot slightly decrease (Fig. S12a, b). As the printing pressure increases, P and D slightly increase (Fig. S12c, d). It can be attributed to the smaller printing speed and larger printing pressure both increasing the thickness of the MXene-CNF layer, resulting in an increase in bending stiffness and less prone to coiling structure. The MXene-CNF/PE bilayer film underwent 15,000 bending cycles without delamination, demonstrating its excellent peel resistance (Fig. S13). The above results provide a solid foundation for the controllable fabrication of ICSBot.

To achieve controllable fabrication, a systematic design framework involving theoretical modeling, FEA, and experimental validation was developed. Key material parameters, including thermal and mechanical properties, were characterized to inform the design process. The anisotropic CTE values of the PE layer were measured as 430 ppm K^−1^ (AD) and 156 ppm K^−1^ (transverse direction, TD), while the MXene-CNF layer exhibited a CTE of − 49 ppm K^−1^ (Fig. S14). Mechanical property analysis revealed that the PE layer had an elastic modulus of 239 MPa, whereas the MXene-CNF layer achieved an elastic modulus of 2.984 GPa, surpassing pure MXene films due to strong hydrogen bonding and its brick-and-tile layered structure (Fig. S15) [44, 45]. To quantitatively describe the coiling structure, the pitch (P) and diameter (D) of the ICSBot were introduced as key parameters (Fig. 2a). Based on laminated composite plate theory, deformation was modeled considering only thickness-dependent parameters (Note S4 and Fig. S16) [46, 47]. For anisotropic laminates, once the mechanical properties are given, the reference curvature tensor ***b*** can be calculated.

1 $$\begin{array}{*{20}c} {\varvec{b} = \left[ {\begin{array}{*{20}c} {\kappa_{1} } & 0 \\ 0 & {\kappa_{2} } \\ \end{array} } \right]} \\ \end{array}$$

where $\kappa_{1}$ and $\kappa_{2}$ are the eigenvalues of the curvature tensor. The maximum value $\kappa_{0}$ in $\kappa_{1}$ and $\kappa_{2}$ is the main curvature, which determines the shape of the ICSBot. The P and D of ICSBot can be calculated by ${\text{D}} = 1/\kappa_{0}$ and ${\text{P}} = 2\pi \tan \theta /\kappa_{0}$. Through theoretical calculations, it has been determined that, given the thickness, elastic modulus, Poisson’s ratio, and CTE of the MXene-CNF and PE layers, the initial coiling structure is predominantly governed by the printing angle *θ* and the temperature variation ΔT. As illustrated in Fig. 2b, when the temperature variation ΔT is held constant (ΔT = 40 °C), the diameter (D) and pitch (P) are solely determined by the printing angle *θ*. The analysis indicates that as *θ* increases, P increases proportionally, while D remains essentially unchanged, as the printing angle does not influence the curvature tensor. For ΔT of 30 °C, the D and P of ICSBot also exhibit similar trends with the variation of printing angle *θ* (Fig. S17). The experimental data and theoretical predictions can be well matched improving the credibility of the theoretical model. When *θ* is fixed at 45°, D and P are exclusively influenced by ΔT. With an increase in ΔT, both P and D decrease, attributable to the greater internal stresses and strain mismatches induced by higher temperature variations (Fig. 2c).

FEA was employed to quantitatively assess the effects of various parameters on the initial coiling structure of the ICSBot (refer to the experimental procedures for further details). Initially, the influence of the dimensions of the rectangular printing pattern, specifically its length (L) and width (W), was examined under fixed *θ* and ΔT conditions. FEA simulations revealed that variations in the pattern length (40, 60, and 80 mm) have no discernible impact on D or P (Fig. S18). Similarly, alterations in the pattern width (2, 4, 6, and 8 mm) do not affect D or P but influence the spacing between adjacent coils (Fig. S19). Notably, when the pattern width exceeds the pitch, overlapping between adjacent coils is observed (Fig. S20). Furthermore, as demonstrated in Fig. S21, for printing angles of ± 45°, the resultant ICSBot structures differ only in coiling direction, with no impact on D or P. To streamline the analysis, subsequent evaluations consider only cases with positive printing angles.

Subsequently, the effects of varying printing angles (*θ* = 15°, 30°, 45°, 60°, and 75°) and temperature variations (ΔT = 20, 30, and 40 °C) on D and P were systematically investigated using FEA (Fig. 2d). Corresponding optical images of the ICSBot are presented in Fig. 2e. The results unequivocally demonstrate that both *θ* and ΔT exert significant influence on the coiling structure. For a fixed *θ* (e.g., 45°), increasing ΔT results in a reduction in both P and D due to elevated thermal stresses (Fig. 2f, g). This trend persists across other printing angles (Fig. S22). Conversely, when ΔT is fixed (e.g., ΔT = 40 °C), increasing *θ* leads to a substantial reduction in P, while D remains largely unaffected (Fig. 2h, i). Analogous trends are observed for ΔT values of 20 and 30 °C (Fig. S23). Besides, the initial structure of ICSBot printed on PE films of different thicknesses (30, 50, and 80 μm) is predicted and experimented (Fig. S24). The results indicate that with the increase of PE layer thickness, both P and D of ICSBot increase. This can be attributed to that thicker PE has greater bending stiffness and is less prone to be bent to produce coiling deformation. The simulation and experimental results have the same trend of change and can be well-matched, substantiating the validity of the proposed model. Furthermore, the FEA methodology facilitates the rapid design of ICSBots with complex initial structures. For instance, ICSBots fabricated with X- and U-shaped printing patterns exhibited complex initial structures that were accurately predicted by the simulations and corroborated by experimental observations (Fig. S25). Programmable ICSBot with complex geometric structures has also been successfully fabricated, exhibiting a biomimetic “ant” structure (Fig. S26). These findings underscore the potential of this FEA-based design framework, integrated with DIW technology, to enable the development of ICSBots with intricate and diverse structural configurations, thereby advancing the next generation of soft robotics. Next, it is necessary to explore the actuation performance of ICSBot to achieve its widespread application.

### Actuation Performance of ICSBot

We evaluated the actuation performance of ICSBot under stimuli including light, humidity, and temperature. First, the photothermal conversion properties of the MXene-CNF layer, which significantly influence the actuation of ICSBot, were analyzed. Figure S27 presents the ultraviolet/visible/near-infrared (UV/Vis/NIR) absorption spectrum of the MXene-CNF layer, indicating its strong absorption in the 400–1000 nm wavelength range. Under different NIR light intensities (100, 200, 300, 400, and 500 mW cm^−2^), the surface temperature of the MXene-CNF layer increased rapidly, stabilizing at 31.61, 37.47, 41.01, 44.56, and 49.51 °C within 3.25 s, demonstrating excellent photothermal conversion efficiency (Fig. S28). Cyclic experiments confirmed the stability of this property. Additionally, the presence of more oxygen-containing functional groups in MXene-CNF can form hydrogen bonds with water molecules. The water contact angle of the MXene-CNF layer is decreased from 71.3° to 53.1°, indicating its excellent water molecule absorption/desorption ability (Fig. S29). To further characterize the absorption/desorption of water molecules, we used FTIR to characterize the MXene-CNF layer after exposure to D_2_O vapor for 1 min (Fig. S30). The vibration absorption peak of D_2_O (~ 2500 cm^−1^) indicates that MXene-CNF layer has the ability to absorb water molecules. The variation of peak intensity at 2500 cm^−1^ with exposure time to air indicates that its absorption of water molecules is reversible. In addition, with the increase in temperature, the MXene-CNF layer undergoes shrinkage (CTE_M*X*ene-CNF_ = −49 ppm K^−1^), and the PE layer undergoes anisotropic expansion with the increase in temperature (CTE_AD_ = 430 ppm K^−1^, CTE_TD_ = 156 ppm K^−1^) (Fig. S14). Based on these properties, including photothermal conversion and thermal shrinkage performance of the MXene-CNF layer, as well as the anisotropic thermal expansion of the PE layer, ICSBot exhibits excellent actuation performance under various stimuli (NIR light, humidity, and temperature).

**Fig. 3:**
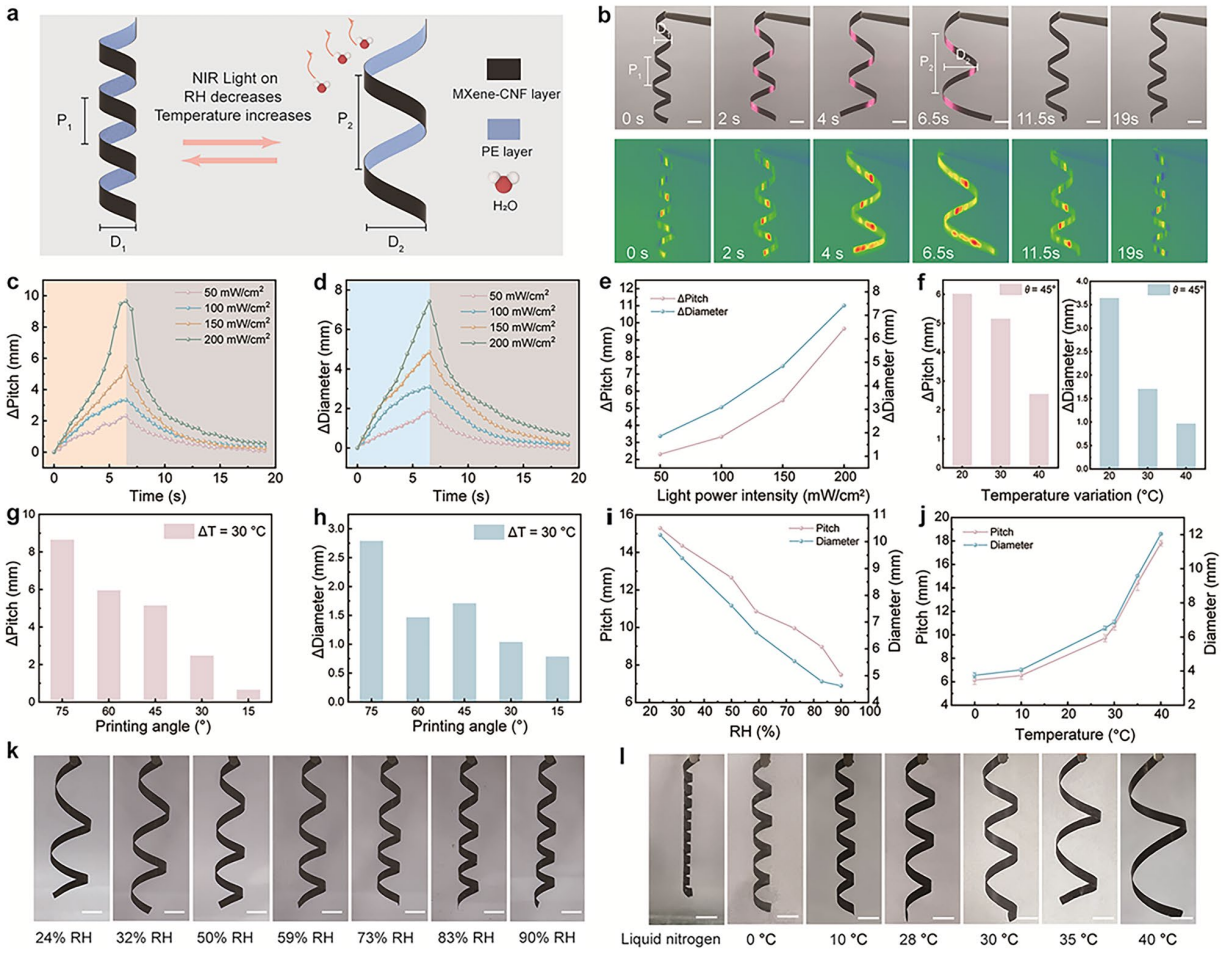
Actuation mechanism and performance of the ICSBot. **a** Schematic actuation mechanisms of ICSBot under NIR light, humidity, and temperature stimuli. **b** Optical and infrared thermal images of the uncoiling deformation of the ICSBot under NIR light (200 mW cm^−2^). **c**, **d** Real-time changes in (**c**) pitch and (**d**) diameter of ICSBot under different NIR light irradiation intensities. **e** Dependence of changes in pitch and diameter of the ICSBot on NIR light irradiation intensities. **f** Dependence of changes in pitch and diameter of the ICSBot on temperature variation under NIR light irradiation (100 mW cm^−2^). **g**, **h** Dependence of changes in (**g**) pitch and (**h**) diameter of the ICSBot on printing angles under NIR light irradiation (100 mW cm^−2^). **i**, **j** Dependence of changes in pitch and diameter of the ICSBot on (**i**) humidity and (**j**) temperature. **k**, **l** Optical images of the coiling deformation of the ICSBot under (**k**) humidity and (**l**) temperature. Scale bars, 5 mm

Figure 3a illustrates the actuation mechanism of ICSBot. Under NIR light irradiation, the MXene-CNF layer converts light energy into heat, leading to a temperature increase. This causes the MXene-CNF layer to shrink due to water molecule desorption, while the PE layer expands due to thermal effects. The resulting strain mismatch induces uncoiling deformation in ICSBot. Upon removal of the light stimulus, the deformation gradually recovers as the temperature decreases, following the reverse process. Similarly, thermal stimulation induces comparable deformation. In contrast, increasing humidity causes the MXene-CNF layer to expand due to water absorption, while the PE layer remains unaffected. This asymmetry in volume change results in coiling deformation.

To achieve controllable deformation of the ICSBot, we quantitatively characterized its actuation performance under various stimulation conditions and intensities. The changes in pitch (ΔP = P_2_ − P_1_) and diameter (ΔD = D_2_ − D_1_) were used as key metrics. As illustrated in Fig. 3b and Movie S1, when the ICSBot (printing angle *θ* = 30°, temperature variation ΔT = 20 °C) is irradiated by an NIR light source (200 mW cm^−2^), both P and D increase simultaneously, with ΔP and ΔD reaching 9.66 and 7.42 mm, respectively, at 6.5 s. Upon switching off the NIR light source, P and D gradually return to their initial values. Thermal infrared imaging reveals that the surface temperature of the ICSBot increases during irradiation and decreases upon cessation, consistent with the deformation and recovery process. As shown in Fig. 3c, d, ΔP and ΔD are fully reversible under varying NIR light intensities, indicating that the uncoiling deformation of the ICSBot is reversible. Furthermore, increasing the NIR light intensity enhances the response speed of the actuator and increases ΔP and ΔD (Fig. 3e), confirming the controllability of the uncoiling deformation. Besides, the actuation performance of ICSBot did not significantly degrade under 80 cycles of NIR irradiation, demonstrating its durability and reliability in practical applications (Fig. S31).

Additionally, the effects of different printing angles (*θ*) and temperature variations (ΔT) on P and D were assessed under constant irradiation time (7 s) and light intensity (100 mW cm^−2^) (Fig. S32). First, we compared the actuation performance of ICSBots fabricated with identical printing angles but varying ΔT. As shown in Fig. 3f, at *θ* = 45°, increasing ΔT results in decreased ΔP and ΔD, indicating that larger ΔT reduces the amplitude of uncoiling deformation. A similar trend is observed for other printing angles (15°, 30°, 60°, and 75°) (Fig. S33). This behavior is likely due to greater internal stress in ICSBots fabricated with higher ΔT, which makes them less responsive to external stimuli. Conversely, when fabricated with a fixed ΔT = 30 °C but varying *θ*, the light-induced ΔP and ΔD increase with larger *θ* values (Fig. 3g, h). These findings demonstrate that ICSBots with different initial structures exhibit varied coiling deformation under identical light intensities, providing versatility for application in diverse environments. The actuation performance of ICSBot under light and humidity stimuli was also evaluated. Figure 3k presents optical images of ICSBot (*θ* = 30°, ΔT = 20 °C) after exposure to different humidity levels for 10 min. At constant humidity, the ICSBot maintains a stable shape. However, as the relative humidity (RH) increases from 24% to 90%, the expansion of the MXene-CNF layer induces tighter coiling deformation. Correspondingly, P decreases from 15.3 to 7.5 mm, and D decreases from 10.2 to 4.6 mm (Fig. 3i). Under thermal stimuli, thermal expansion of the PE layer leads to uncoiling deformation, as shown in the optical images in Fig. 3l. With temperature increases from 0 to 40 °C, P increases from 6.1 to 17.8 mm, and D increases from 3.8 to 12 mm (Fig. 3j). As shown in Fig. S34, with the continuous increase of temperature, the coiling structure of ICSBot gradually unfolds completely (50 °C), and then produces the opposite coiling structure of MXene-CNF layer inside and PE layer outside (60, 70, and 80 °C). Notably, under exposure to liquid nitrogen vapor, ICSBot undergoes significant shrinkage deformation, with reductions in P and D of 78.1% and 76.7%, respectively, demonstrating its potential for applications in extreme low-temperature environments.

### Snake-Inspired Coiling Grasping

Smart grippers, characterized by their simple structure, high degrees of freedom, and exceptional flexibility, are widely utilized in robotics [48, 49]. However, most existing smart grippers consist of four or more bendable arms that contact different points on an object to provide grasping force upon stimulation. These grippers often have limited contact areas with objects, making it challenging to grasp round, slender, or irregularly shaped items. Therefore, designing soft grippers capable of establishing broader contact areas, enhancing contact friction, and effectively grasping objects of diverse shapes remains a critical objective.

**Fig. 4:**
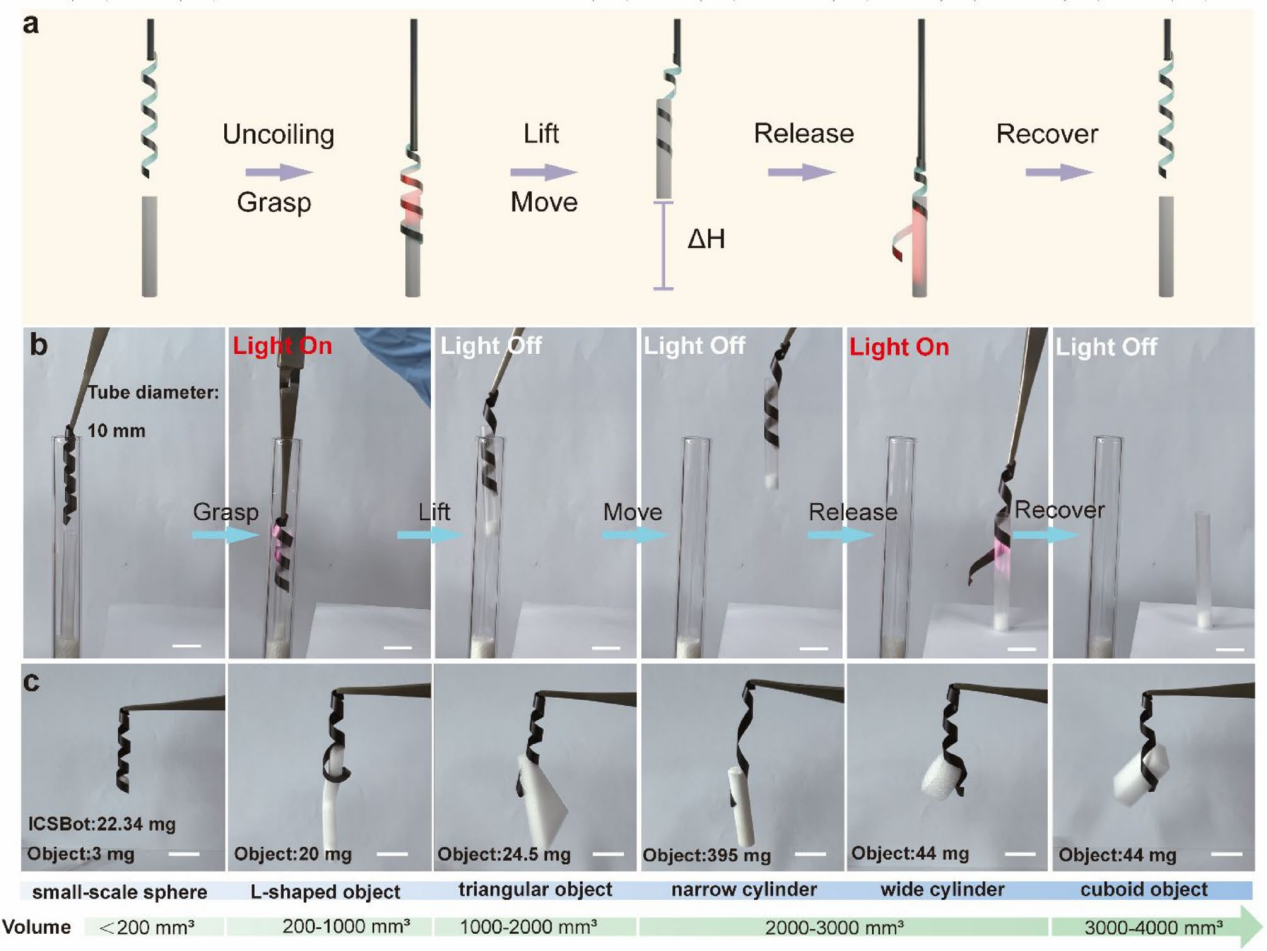
Snake-inspired coiling gripper under NIR light illumination. **a** Schematic diagram of the grasping process of coiling gripper. **b** Optical image of the process of coiling gripper grasping objects in a narrow tube. **c** Optical image of coiling gripper grasping different objects. Scale bars, 10 mm

Drawing inspiration from snake prey-handling mechanisms, we developed a coiling gripper based on the ICSBot. As illustrated in Fig. 4a, the ICSBot, initially in a coiled state, gradually uncoils under NIR light irradiation and moves to the exterior of the target object. Upon cessation of the light stimulus, the ICSBot returns to its original coiled configuration, firmly locking around the object. The ICSBot can move objects and release them when re-illuminated with light. Unlike conventional soft robotic grippers with four or more arms, the ICSBot tightly coils around the object, significantly increasing the contact friction and expanding the applicability of the gripper to various object geometries. Additionally, in contrast to other light-driven grippers, this coiling approach enables continuous object grasping even after the stimulus is removed, thereby reducing energy consumption. With its compact size and firm grasping capabilities, the ICSBot is particularly suited for operations in confined spaces. Figure 4b and Movie S2 demonstrate the process of grasping a slender cylindrical object within a narrow tube. Initially, the ICSBot is positioned inside a 10 mm diameter tube. Under NIR light irradiation, it uncoils and moves to the exterior of the object. Upon removal of the light source, the ICSBot tightly grips the object, enabling its removal from the tube. The target object is subsequently released upon re-irradiation. Furthermore, the ICSBot is capable of grasping objects of various shapes, including L-shaped items, cylinders, spheres, triangular prisms, and cuboids (Fig. 4c). It is necessary to optimize NIR light irradiation area for grasping different objects to improve grasping efficiency. For large objects, we irradiate ICSBot to produce uncoiling deformation until it is enough to wrap large objects. For small-sized objects, we can locally illuminate the ICSBot to improve grasping efficiency. Objects ranging in volume from 179 mm^3^ (sphere) to 3600 mm^3^ (cuboid) can be securely grasped and lifted, showcasing the stability and versatility of the coiling grasping.

### Snake-Inspired Multimodal Locomotion

Crawling locomotion, one of the most common modalities of soft robots, has been extensively studied and applied [50–52]. Snakes, with their diverse locomotion modes, including sidewinding, accordion crawling, and winding climbing, are capable of navigating complex and challenging environments such as deserts, grasslands, and forests. This versatility makes snake-inspired locomotion an area of significant research interest. However, replicating multimodal locomotion in soft robots remains a substantial challenge.

#### Snake-Inspired Sidewinding Crawling Locomotion

**Fig. 5:**
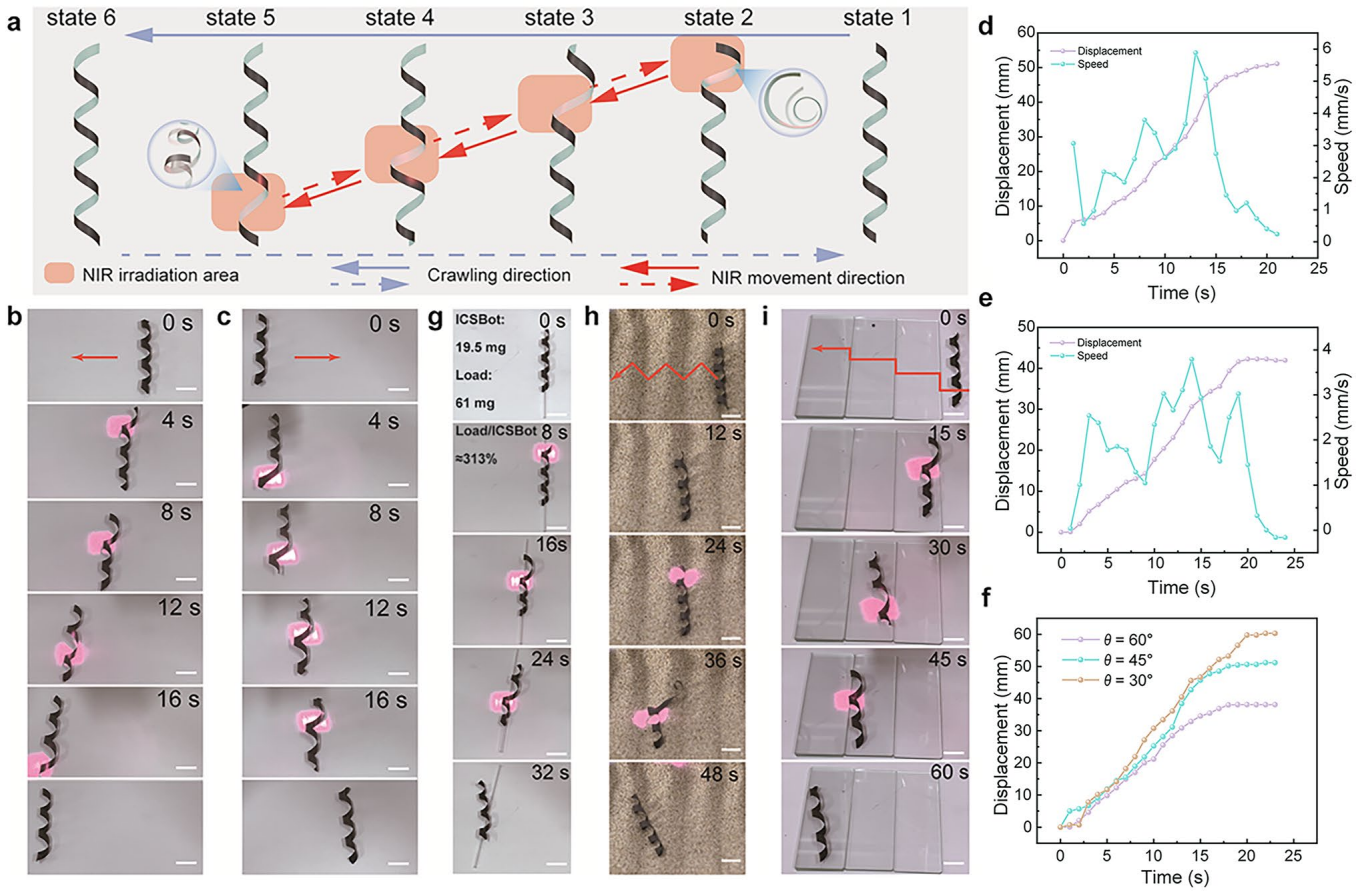
Snake-inspired sidewinding crawling locomotion under NIR light illumination. **a** Schematic diagram of the sidewinding crawling process. **b**, **c** Optical images of forward and backward sidewinding crawling process. **d**, **e** Displacement evolvement with time during the forward and backward sidewinding crawling locomotion. **f** Displacement evolvement with time of ICSBot at different printing angles (*θ* = 30°, 45°, and 60°). **g** Crawling under load conditions. **h**, **i** Crawling in an unstructured environment. Scale bars, 10 mm

Inspired by the sidewinding locomotion of snakes in harsh environments, we developed an ICSBot capable of crawling in a manner similar to snakes (Fig. 5a). Under NIR light irradiation on the top of the ICSBot, uncoiling deformation occurs until the coiling structure contacts the ground (state 2). This shifts the center of gravity forward, generating a torque that drives the robot to crawl. As the NIR light source moves along the spiral unfolding line, the coiling structure entering the irradiation range begins to uncoil and deform (state 3), while previously deformed areas gradually recover (state 4). The generation of the driving force is due to the gradual displacement of the center of gravity caused by the continuous uncoiling deformation at different positions, resulting in continuous forward crawling. Finally, when the light irradiation area is moved to the end of the ICSBot (state 5), the robot completes a locomotion cycle. Reversing the light direction enables crawling in the opposite direction (Fig. 5b, c and Movie S3). Figure 5d, e illustrates the displacement and speed of ICSBot during forward and backward crawling under NIR irradiation, achieving maximum displacements of 51.1 and 42.3 mm, respectively. Uneven crawling speed is caused by the accumulation of energy required to overcome the maximum torque during the uncoiling process. Experiments reveal that the size of the ICSBot significantly influences locomotion. ICSBots with lengths exceeding 100 mm or widths greater than 6 mm experience excessive friction, hindering crawling. Optimal locomotion was achieved with a rectangular pattern size of 80 mm × 4 mm. Additionally, crawling displacement is influenced by the printing angle (*θ*) during programmed NIR irradiation (Figs. 5f and S35). Smaller printing angles result in longer unfolding lines, enabling greater displacement. These findings demonstrate that both the crawling direction and distance can be precisely controlled via light source direction and printing angle. To ensure the accuracy of robot movement, the path and intensity of NIR light are unified during each irradiation process. It is worth noting that optimizing the NIR light path is beneficial for improving the motion efficiency of ICSBot. Using the contact point between one end of the ICSBot and the ground as the starting point for NIR irradiation can effectively reduce lag effects and energy loss. The response speed of ICSBot can be improved by increasing the intensity of NIR light irradiation.

Moreover, ICSBot demonstrates continuous crawling under load and in unstructured environments. As shown in Fig. 5g and Movie S4, ICSBot carrying a load of 313% of its weight achieved a displacement of 35.3 mm in 32 s under NIR irradiation. The robot also successfully traversed wavy sandy terrain and stair-like obstacles (Fig. 5h, i and Movies S5, S6). In complex environments, ICSBot can escape from complex environments and propel objects forward (Fig. S36 and Movies S7, S8). The motion accuracy and stability of ICSBot in high-noise environments are crucial for its application in practical scenarios [53]. Here, ICSBot is still able to stably crawl in a high-noise environment (xenon lamp, 60 mW cm^−2^) with sidewinding locomotion under NIR light irradiation (Fig. S37 and Movie S9). It can be attributed to the fact that overcoming friction requires sufficient energy input, and the deformation of ICSBot caused by noisy environments is relatively small. These results highlight the robust crawling capabilities of ICSBot, providing a novel approach for untethered robots navigating unstructured environments.

#### Snake-Inspired Accordion Crawling Locomotion

**Fig. 6:**
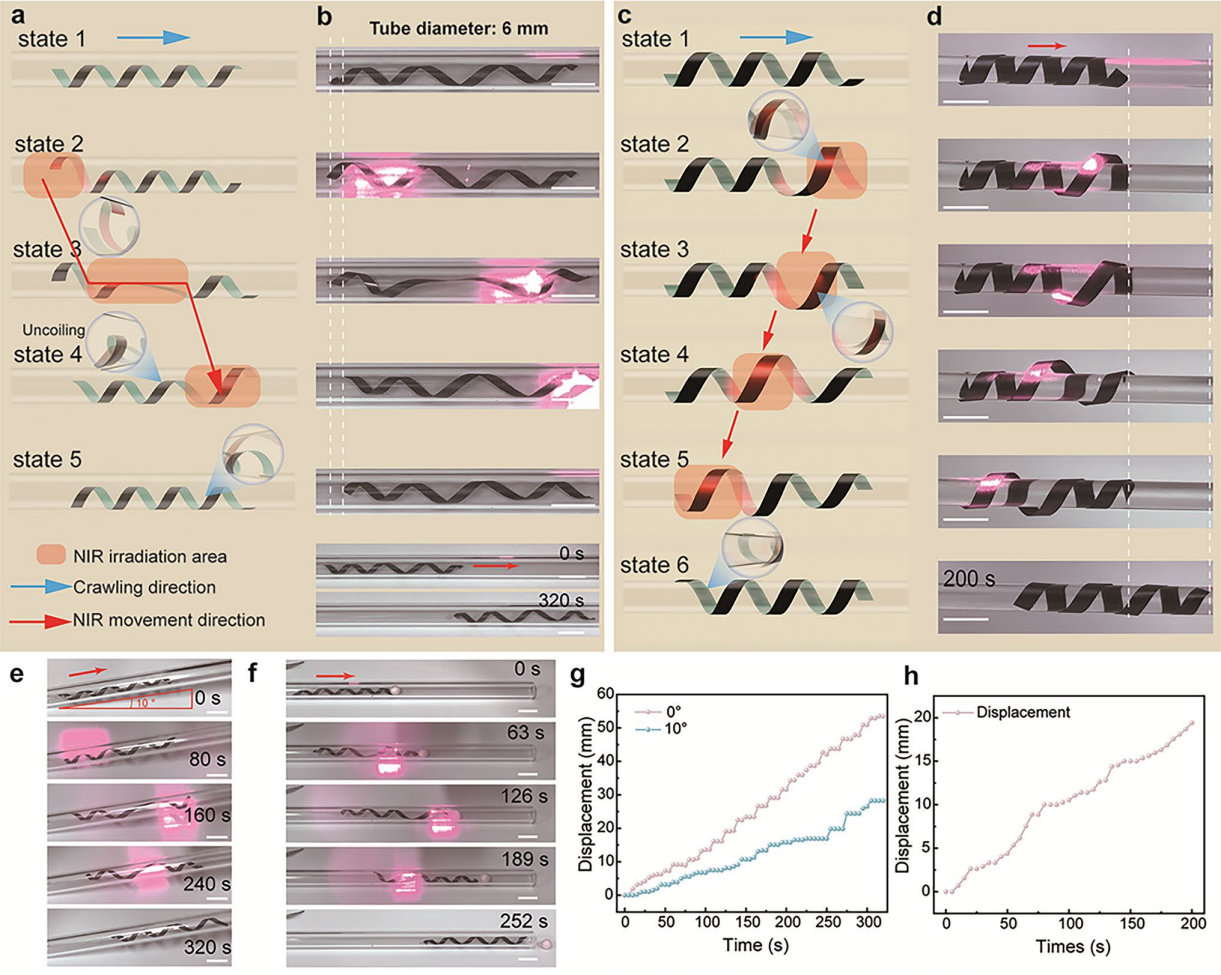
Snake-inspired accordion crawling and winding climbing locomotion under NIR light illumination. **a**, **b** Schematic diagram (**a**) and optical images (**b**) of the accordion crawling process under programmed NIR light irradiation. **c**, **d** Schematic diagram (**c**) and optical images (**d**) of the winding climbing process under programmed NIR light irradiation. **e** Accordion crawling inside the inclined tube. **f** Accordion crawling under the condition of pushing objects. **g** Displacement evolvement with time during the accordion crawling locomotion. **h** Displacement evolvement with time during the winding climbing locomotion. Scale bars, 10 mm

To address the challenges of narrow-space navigation, we developed an ICSBot capable of accordion-style crawling under NIR light stimulation. Figure 6a, b depicts the operation of ICSBot inside a tube. Accordion crawling involves four sequential steps. Firstly, NIR light is irradiated onto the back end of the ICSBot, and the coiling structure on the back end is uncoiled until it is tightly attached to the inner wall of the tube (state 2). Next, the NIR light source is slowly moved forward along the tube. Due to the frictional force between the back end of the ICSBot and the tube, the coiling structure in the middle part uncoils to the front, causing the robot to elongate to the front inside the tube (state 3). Then the NIR light source is irradiated on the front end of ICSBot, and the coiling structure on the front end uncoils until it is tightly attached to the inner wall of the tube (state 4). At this point, the deformation of the coiling structure at the backside begins to recover, and the friction with the tube decreases. Influenced by the friction between the front side coiling structure and the tube wall, the ICSBot contracts to the front. Finally, after the NIR light is removed, the deformation of the ICSBot is recovered, resulting in an overall forward displacement (state 5). During NIR irradiation, ICSBot moves approximately 3.4 mm per cycle. The key to accordion crawling lies in the controlled friction shifts and uncoiling deformation, enabling efficient locomotion even in confined spaces. In a 6 mm diameter tube, the robot achieved a forward displacement of 53.4 mm within 320 s (Fig. 6g and Movie S10). It also performed well in inclined tubes (10°), with a displacement of 28.2 mm in 320 s (Fig. 6e, g and Movie S11). Additionally, ICSBot can push a spherical object within the tube, displacing it by 65.7 mm in 252 s (Fig. 6f and Movie S12). The key to accordion crawling lies in the controlled friction shifts and uncoiling deformation, enabling efficient locomotion even in confined spaces. In S-shaped and U-shaped rubber tubes, ICSBot can still navigate smoothly, which demonstrates the stability of ICSBot in practical application scenarios of dynamic obstacles (Fig. S38 and Movies S13, S14).

#### Snake-Inspired Winding Climbing Locomotion

Inspired by the climbing trees of snakes, we designed an ICSBot capable of climbing along the outer wall of a tube under NIR light stimulation. As shown in Fig. 6c, d, the climbing process consists of six states: (1) ICSBot is placed outside the tube. (2) NIR light irradiates the front end, uncoiling it away from the tube surface. (3) Friction from the remaining coiled structure prevents backward movement, enabling forward extension. (4) As the light moves, previously deformed areas recover, fixing the ICSBot in a forward position. (5) The back end uncoils, reducing friction. (6) Recovery of the back end completes the climbing cycle, allowing periodic movement under NIR irradiation. As demonstrated in Fig. 6h and Movie S15, ICSBot climbed 19.4 mm along the outer wall of a 6 mm diameter tube within 200 s. This winding climbing capability expands the application potential of ICSBot for locomotion in unstructured environments. In addition, ICSBot can climb along the outer wall of trembling rubber tubes, further demonstrating its ability to apply in dynamic obstacle environments (Fig. S39 and Movie S16).

## Conclusions

Inspired by the diverse locomotion modes of snakes, ICSBot was fabricated by printing MXene-CNF ink onto pre-expanded PE film using DIW technology. Leveraging the anisotropic deformation properties of the PE film, the bilayer structure achieves a controllable initial coiling shape upon recovery to room temperature. The fabrication of ICSBot was guided by theoretical calculations and finite element analysis, enabling precise prediction and optimization of its initial structure. The robot demonstrates reversible coiling deformation under various stimuli (NIR light, humidity, temperature), based on the photothermal conversion and moisture absorption/desorption properties of the MXene-CNF layer and the anisotropic thermal expansion of the PE layer. Building on these features, the ICSBot achieves four biomimetic locomotion modes: (1) grasping inspired by snake prey handling, enabling secure object manipulation in confined spaces; (2) sidewinding crawling, facilitating directional and load-bearing motion in unstructured environments; (3) accordion crawling, allowing traversal through narrow tubes and internal object extraction; and (4) winding climbing, enabling locomotion along tubular surfaces. Compared with other reported soft robots (snake-like robot, coiling structure soft robot, multimodal locomotion soft robot), our ICSBot shows more excellent flexibility, more stimulus–response abilities, and more locomotion modes (Table S2). This study highlights the potential of combining finite element analysis with DIW technology to develop soft robots with tunable coiling structures. The snake-inspired multimodal locomotion capabilities demonstrated by ICSBot offer new avenues for designing smart and multifunctional soft robotic systems.

## Supplementary Information

Below is the link to the electronic supplementary material.

Supplementary file1 (DOCX 7618 KB)

Supplementary file2 (MP4 1124 KB)

Supplementary file3 (MP4 931 KB)

Supplementary file4 (MP4 410 KB)

Supplementary file5 (MP4 298 KB)

Supplementary file6 (MP4 719 KB)

Supplementary file7 (MP4 489 KB)

Supplementary file8 (MP4 759 KB)

Supplementary file9 (MP4 277 KB)

Supplementary file10 (MP4 413 KB)

Supplementary file11 (MP4 1122 KB)

Supplementary file12 (MP4 1252 KB)

Supplementary file13 (MP4 1210 KB)

Supplementary file14 (MP4 1846 KB)

Supplementary file15 (MP4 1598 KB)

Supplementary file16 (MP4 646 KB)

Supplementary file17 (MP4 1085 KB)
