# Supplementary material for: From Coils to Crawls: A Snake-Inspired Soft Robot for Multimodal Locomotion and Grasping
Source: Nanomicro Lett. 2025 Apr 30;17:243. doi: 10.1007/s40820-025-01762-9 (PMC12043558; doi:10.1007/s40820-025-01762-9)
Supplement: Supplementary file 1 — Supplementary file1 (DOCX 7618 KB) [file 40820_2025_1762_MOESM1_ESM.docx]

Supporting Information for

**From Coils to Crawls: A Snake-Inspired Soft Robot for Multimodal Locomotion and Grasping**

He Chen^1^, Zhong Chen^2,^*, Zonglin Liu^1^, Jinhua Xiong^1^, Qian Yan^1^, Teng Fei^1^, Xu Zhao^1^, Fuhua Xue^1^, Haowen Zheng^1^, Huanxin Lian^1^, Yunxiang Chen^1^, Liangliang Xu^1^, Qingyu Peng^1,3,^*, and Xiaodong He^1,^*

^1^ National Key Laboratory of Science and Technology on Advanced Composites in Special Environments, Center for Composite Materials and Structures, Harbin Institute of Technology, Harbin 150080, P. R. China

^2^ Dongfang Electric Academy of Science and Technology Co., Ltd, Chengdu 611731, P. R. China

^3^ Suzhou Research Institute of HIT, Suzhou 215104, P. R. China

*Corresponding authors. E-mail: [pengqingyu@hit.edu.cn](mailto:pengqingyu@hit.edu.cn) (Qingyu Peng); [nick.chenzhong@outlook.com](mailto:nick.chenzhong@outlook.com) (Zhong Chen); [hexd@hit.edu.cn](mailto:hexd@hit.edu.cn) (Xiaodong He)

**S1 Characterization**

The morphology of MXene nanosheets, MXene-CNF layer surface, and MXene-CNF/PE bilayer film cross-section were recorded by scanning electron microscopy (FIB-SEM, TESCAN AMBER) with an energy dispersive spectrometer (EDS) detector.

The thickness of MXene nanosheets was recorded by atomic force microscopy (AFM, Dimension Fastscan, Brucker.).

The structures of MXene and MXene-CNF were characterized using Fourier transform infrared spectroscopy (FT-IR, Thermo Scientific Nicolet iS20), X-ray photoelectron spectroscopy (XPS, ESCALAB 250Xi), and X-ray diffraction (XRD, Bruker D8 Advance).

The water contact angles of PE film before and after plasma treatment, as well as the water contact angles of MXene, CNF, and MXene-CNF, were measured using a contact angle (Datophysics DCAT21).

The optical absorption spectrum of CNF and MXene-CNF were measured using a UV/Vis/IR spectrometer (Shimadzu UV-3600i Plus).

The rheological properties of MXene ink and MXene-CNF ink were measured using flat plate rheometer (TA Instruments DHR-3).

The mechanical properties of MXene film, MXene-CNF film, and PE film were measured using a mechanical tensile machine (Instron 5944).

The thermal shrinkage performance of MXene-CNF film and the thermal expansion performance of anisotropic PE film were measured by a thermomechanical analyzer (TMAQ400).

The photothermal conversion performance of MXene-CNF film and the light-driven actuation performance of ICSBot were evaluated using an 808 nm NIR laser, and the NIR light intensity was measured using an optical power meter (Ceaulight, GEL-NP2000). The infrared thermal images are recorded with a VARIOCAM HD infrared camera (Infratech).

The humidity-driven actuation performance of the ICSBot was measured in an airtight chamber. The relative humidity was controlled by using saturated aqueous solutions of CH_3_COOK, MgCl_2_, K_2_CO_3_, NaBr, NaCl, KCl, and K_2_SO_4_ in an enclosed container, which generated ∼23%, 33%, 44%, 57%, 75%, 86%, and 97% RH, respectively. Besides, the RH value was recorded with a hydrometer.

**S2 Supplementary Notes**

**Note S1** The single layer of MXene was obtained by a modified HCl/LiF etching method (**Fig. S1**). **Figure S1a** shows the X-ray diffraction (XRD) patterns of the obtained Ti_3_C_2_T_x_ MXene and Ti_3_AlC_2_ MAX phase, the characteristic diffraction peak corresponding to the (002) lattice plane of Ti_3_C_2_T_x_ MXene is 6.59°, which is significantly smaller than that of Ti_3_AlC_2_ (9.67°). The characteristic diffraction peak at 38.89° corresponds to the (104) lattice plane of Ti_3_AlC_2_, which disappears in the XRD pattern of Ti_3_C_2_T_x_ MXene, indicating that the aluminum atomic layers have been selectively etched [S1]. The SEM image (**Fig. S1b**) and atomic force microscopy (AFM) image (**Fig. S1c**) indicate the successful synthesis of single-layer MXene.

**Note S2** MXene-CNF ink was successfully fabricated for DIW by uniformly mixing the concentrated MXene dispersion with CNF. The obvious Tyndall effect indicates the uniform dispersion of MXene-CNF ink (**Fig. S2**). The crystal structure, intermolecular hydrogen bonding, and chemical composition of MXene-CNF inks were analyzed using XRD, FT-IR spectroscopy, and XPS (**Fig. S3**). The XRD spectra of MXene, CNF, and MXene CNF are shown in **Fig. S3a**. The introduction of CNF shifts the characteristic peak of the (002) crystal plane of MXene from 6.59° to 5.94°, indicating that the introduction of 1D CNF further expands the interlayer spacing of 2D MXene flakes, promotes the adsorption/desorption of water molecules, and effectively avoids the re-stacking of MXene nanosheets. The FT-IR spectrum is shown in **Fig. S3b**, the CNF in MXene-CNF shows typical peaks at 2931 cm^-1^ and 1644 cm^-1^, corresponding to C–H stretching and –OH bending, respectively. The distinctive peaks of MXene in MXene-CNF are 1428 and 580 cm^-1^ for C–F and –OH groups, respectively. The XPS spectrum further confirms the presence of a large number of oxygen-containing functional groups in MXene-CNF (**Fig. S3c, d**).

**Note S3** The rheological properties of MXene-CNF ink are the key factors determining the successful fabrication of ICSBot through DIW technology [S2]. Specifically, MXene-CNF ink obviously presents a low-viscosity gel state (**Fig. S4a**). As the shear rate increases (from 10^-2^ s^-1^ to 10^3^ s^-1^), MXene-CNF inks with different solid contents exhibit shear thinning non-Newtonian fluid behavior, which ensures continuous and smooth flow of ink at the nozzle, thereby ensuring its extrusiveness (**Fig. S4b**). As the solid content increases, the viscosity of MXene-CNF ink also increases, indicating that adjusting the solid content can easily and effectively control the ink viscosity. Under the same solid content, MXene-CNF ink has a much higher viscosity than MXene ink, indicating that the addition of CNF forms a strong encapsulation network with MXene. Furthermore, the thixotropy of ink was studied through alternating changes in shear rate (0.1 s^-1^/100 s^-1^) (**Fig. S4c**). With the alternation of shear rate from 0.1 s^-1^ to 100 s^-1^, the gel network of MXene-CNF ink was destroyed, and the viscosity of gel with different solid content decreased rapidly. After the shear rate recovered to 0.1 s^-1^, the viscosity of the ink quickly returned to its initial value, indicating that the ink extruded from the nozzle has the ability to maintain a stable shape. In summary, the excellent rheological properties of MXene-CNF provide a solid foundation for the fabrication of ICSBot using DIW technology.

**Note S4** We calculated the overall deformation of ICSBot using laminated composite plates theory, with only thickness being involved in the geometric parameters [S3, S4].

The generation of deformation can be attributed to the stress generated by thermal expansion, and its constitutive relationship can be expressed as:

$$\begin{aligned} \sigma=\sum_{i=1}^{2} \boldsymbol{D}_{\boldsymbol{i}}\left（ \varepsilon-\varepsilon_{ti} \right）\#\left( S1 \right) \end{aligned}$$

Where$i=1$ represents the PE layer and$i=2$ represents the MXene-CNF layer, $\boldsymbol{D}_{\boldsymbol{i}}$ and $\varepsilon_{ti}$ represent the material stiffness and thermal strain of the i-th layer, respectively. $\sigma$is the stress and $\varepsilon$ is the strain.

The z-direction is defined as the thickness direction of the bilayer film. It is possible to describe the total strain at distance z from the bottom plane ($z_{0}=0$) as $\varepsilon(z)=\varepsilon_{0}+\boldsymbol{\kappa}z$ under Kirchoff assumption, where $\varepsilon_{0}$ is the bottom plane strain and $\kappa$ the curvature. The forces and moments can be expressed as:

$$\begin{aligned} \boldsymbol{F}=\boldsymbol{A}\epsilon_{0}+\boldsymbol{B} \kappa\#(S2) \end{aligned}$$

$$\begin{aligned} \boldsymbol{M}=\boldsymbol{B}\epsilon_{0}+\boldsymbol{C} \kappa\boldsymbol{\#}\left( S3 \right) \end{aligned}$$

where ***F*** and ***M*** are the force matrix and moment matrix, respectively. ***A, B*** and ***C*** are the extensional stiffness, coupling stiffness and bending stiffness, respectively, which defined as:

$$\begin{aligned} \boldsymbol{A}=\sum_{k=1}^{2} \boldsymbol{D}_{k}\left( z_{k+1}-z_{k} \right)\boldsymbol{\#}\left( S4 \right) \end{aligned}$$

$$\begin{aligned} \boldsymbol{B}=\frac{1}{2}\sum_{k=1}^{2} \boldsymbol{D}_{k}\left( z_{k+1}^{2}-z_{k}^{2} \right)\boldsymbol{\#}\left( S5 \right) \end{aligned}$$

$$\begin{aligned} \boldsymbol{C}=\frac{1}{3}\sum_{k=1}^{2} \boldsymbol{D}_{k}\left( z_{k+1}^{3}-z_{k}^{3} \right)\boldsymbol{\#}\left( S6 \right) \end{aligned}$$

where k is the k-th layer definition and **D** is the stiffness matrix defined as:

$$\begin{aligned} \boldsymbol{D}=RH^{-1}R^{-1}\left( \begin{matrix} \frac{E_{11}}{1-\upsilon_{12}\upsilon_{21}} & \frac{E_{11}\upsilon_{21}}{1-\upsilon_{12}\upsilon_{21}} & 0 \\ \frac{E_{22}\upsilon_{12}}{{1-\upsilon}_{12}\upsilon_{21}} & \frac{E_{22}}{{1-\upsilon}_{12}\upsilon_{21}} & 0 \\ 0 & 0 & G_{12} \end{matrix} \right)\boldsymbol{\#}\left( S7 \right) \end{aligned}$$

$$=\frac{E}{1-\upsilon^{2}}\left( \begin{matrix} 1 & \upsilon& 0 \\ \upsilon& 1 & 0 \\ 0 & 0 & \frac{1-\upsilon}{2} \end{matrix} \right)$$

where the PE layer and MXene-CNF layer are assumed to be isotropic with E and ν being respectively Young’s modulus and Poisson’s ratio, and ***H*** is transformation matrix, ***R*** is Reuter's matrix, where *θ* is the printing angle:

$$\begin{aligned} \boldsymbol{H}=\left( \begin{matrix} \cos^{2} \theta& \sin^{2} \theta& 2\sin\theta\cos\theta\\ \sin^{2} \theta& \cos^{2} \theta& -2\sin\theta\cos\theta\\ -sin \theta\cos\theta& \sin\theta\cos\theta& \cos^{2} \theta-\sin^{2} \theta\end{matrix} \right)\boldsymbol{\#}\left( S8 \right) \end{aligned}$$

$$\begin{aligned} \boldsymbol{R}=\left( \begin{matrix} 1 & 0 & 0 \\ 0 & 1 & 0 \\ 0 & 0 & 2 \end{matrix} \right)\boldsymbol{\#}\left( S9 \right) \end{aligned}$$

Since deformation is induced by thermal expansion, the total strain can be modeled according to the definition of CTE, which represents the amount of strain generated per percentage unit of temperature change.

The thermal stress can be expressed as $\boldsymbol{\epsilon}_{\boldsymbol{t}}=\boldsymbol{\alpha} \Delta T= \boldsymbol{\alpha} (T- T_{0})$, where 𝜶 is the linear CTE, T is the pre-heating platform temperature at which the bilayer film shows a curvature 𝜿 = 0 and T_0_ is the room temperature.

$$\begin{aligned} \boldsymbol{\alpha}=\left( \begin{matrix} \alpha_{xx} \\ \alpha_{yy} \\ \alpha_{xy} \end{matrix} \right)=\boldsymbol{R}\boldsymbol{H}^{\boldsymbol{-1}}\boldsymbol{R}^{\boldsymbol{-1}}\left( \begin{matrix} \alpha_{1} \\ \alpha_{2} \\ \alpha_{0} \end{matrix} \right)\boldsymbol{\#}\left( S10 \right) \end{aligned}$$

Next, **F** and **M** can be represented as:

$$\begin{aligned} \boldsymbol{F}=\int_{0}^{h} \boldsymbol{D} \boldsymbol{\alpha} \Delta T dz\boldsymbol{\#}\left( S11 \right) \end{aligned}$$

$$\begin{aligned} \boldsymbol{M}=\int_{0}^{h} \boldsymbol{D} \boldsymbol{\alpha} \Delta T z dz\boldsymbol{\#}\left( S12 \right) \end{aligned}$$

where h is the whole thickness of the bilayer film and ΔT is the temperature variations.

Since **D** and **α** are independent of z-axis, **F** and **M** can be written as:

$$\begin{aligned} \boldsymbol{F}=\sum_{k=1}^{2} \boldsymbol{D}_{\boldsymbol{k}} \boldsymbol{\alpha}_{\boldsymbol{k}} \Delta T \left( z_{k+1}-z_{k} \right)\boldsymbol{\#}\left( S13 \right) \end{aligned}$$

$$\begin{aligned} \boldsymbol{M}=\frac{1}{2}\sum_{k=1}^{2} \boldsymbol{D}_{\boldsymbol{k}}\boldsymbol{\alpha}_{\boldsymbol{k}} \Delta T \left( z_{k+1}^{2}-z_{k}^{2} \right)\boldsymbol{\#}\left( S14 \right) \end{aligned}$$

The strain and the curvature vector can be calculated using the definition of **F** and **M**:

$$\begin{aligned} \left( \begin{matrix} \boldsymbol{\varepsilon}_{\boldsymbol{0}} \\ \boldsymbol{\kappa} \end{matrix} \right)=\left( \begin{matrix} \boldsymbol{A} & \boldsymbol{B} \\ \boldsymbol{B} & \boldsymbol{C} \end{matrix} \right)^{-1}\left( \begin{matrix} \boldsymbol{F} \\ \boldsymbol{M} \end{matrix} \right)\#\left( S15 \right) \end{aligned}$$

The curvature vector $\boldsymbol{\kappa}={(\kappa_{xx} \kappa_{yy} \kappa_{xy})}^{T}$ can be transformed in the matrix form:

$$\begin{aligned} \boldsymbol{b}=\left( \begin{matrix} \kappa_{xx} & \kappa_{xy} \\ \kappa_{xy} & \kappa_{yy} \end{matrix} \right)\boldsymbol{\#}\left( S16 \right) \end{aligned}$$

From Kirchoff’s theory of thin plates, the maximum eigenvalue of the curvature tensor **b** represents the principal curvature 𝜅_0_, which determines the shape of ICSBot.

Finally, the diameter D and the pitch P of the ICSBot are finally calculated as:

$$\begin{aligned} D=\frac{2}{\kappa_{0}}\#\#\left( S17 \right) \end{aligned}$$

$$\begin{aligned} P=\frac{2\pi\tan\theta}{\kappa_{0}}\#\left( S18 \right) \end{aligned}$$

**S3 Supplementary Figures and Tables**


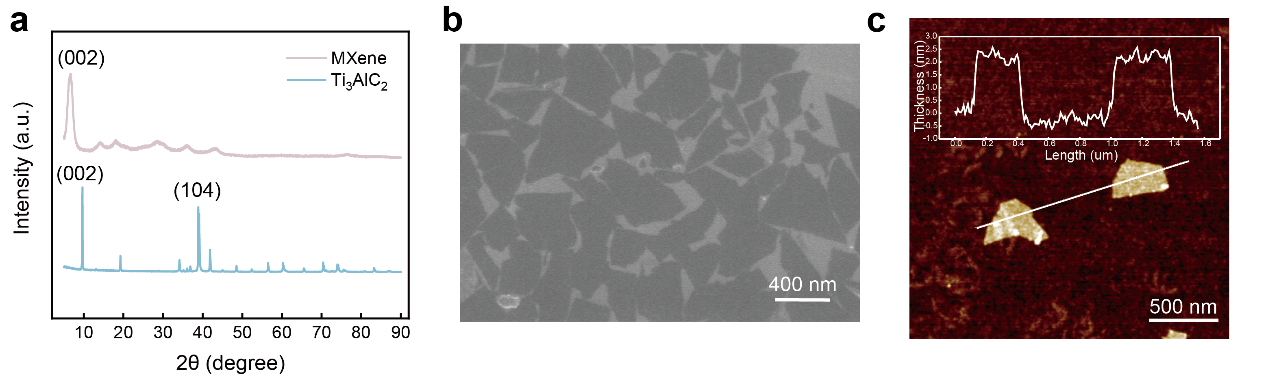


**Fig. S1** Characterization of single-layer MXene. **a** XRD patterns of Ti_3_AlC_2_ and MXene, **b** SEM image of single-layer MXene, **c** AFM image of single-layer MXene

**
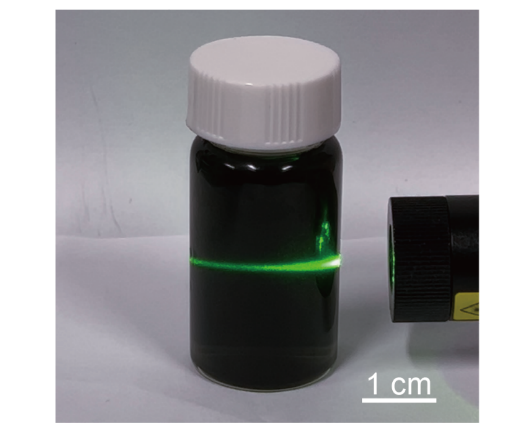
**

**Fig. S2** Optical image of the stability of MXene-CNF ink


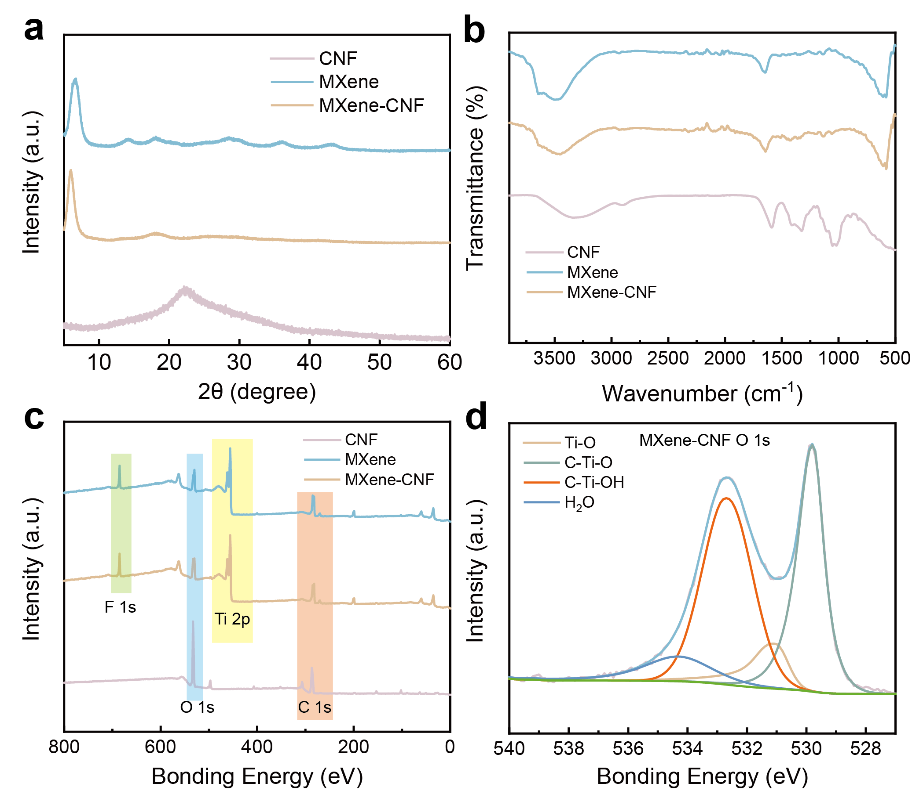


**Fig. S3** Characterization of MXene-CNF layer. **a** XRD patterns of MXene, CNF, and MXene-CNF, **b** FT-IR spectra of MXene, CNF, and MXene-CNF, **c** XPS spectra of MXene, CNF, and MXene-CNF, **d** O1s XPS spectra of MXene-CNF


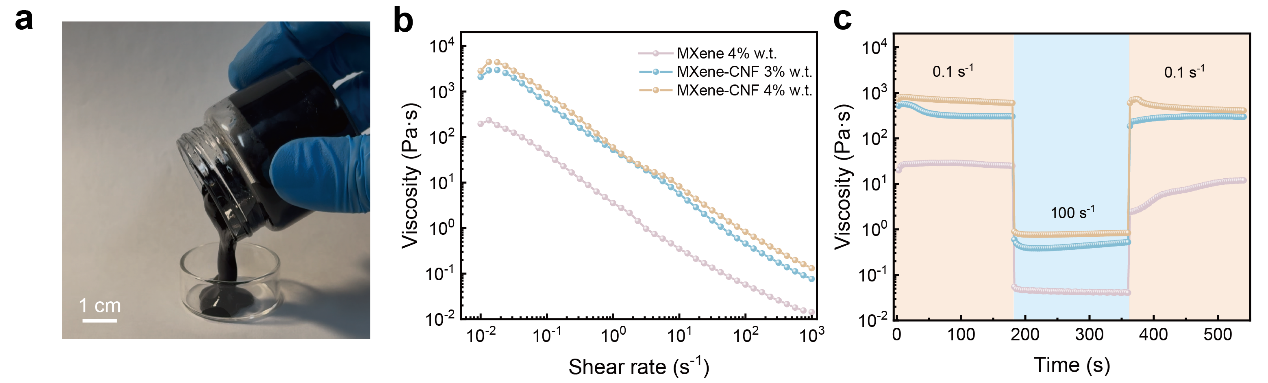


**Fig. S4** The rheological properties of MXene-CNF ink. **a** Optical image of MXene-CNF ink, **b** Viscosity as a function of shear rate, **c** Viscosity evolution over time for alternating shear rates of 0.1 s^−1^ and 100 s^−1^


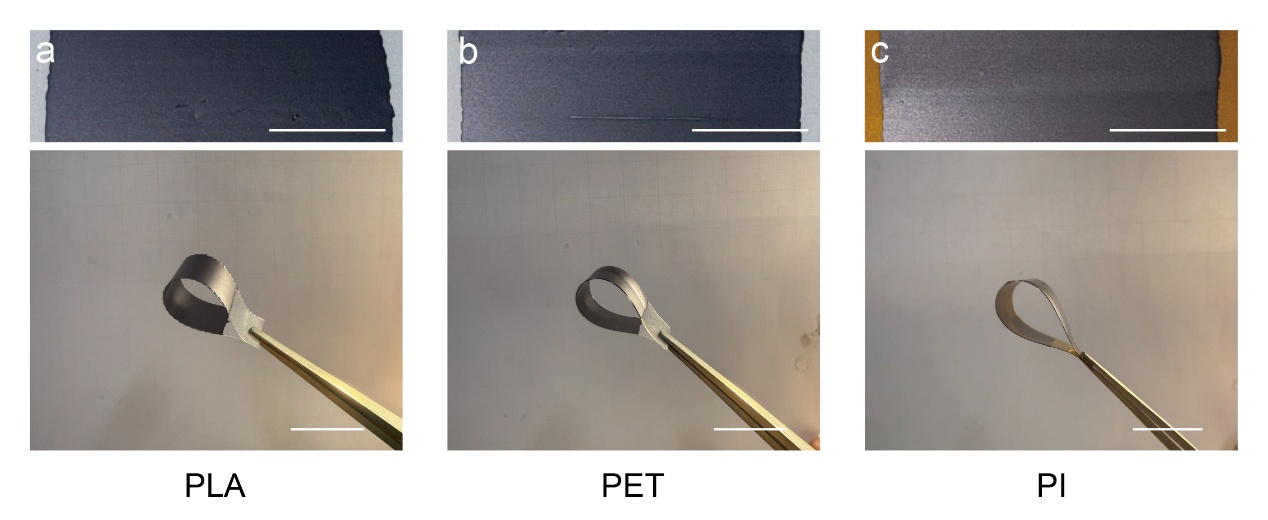


**Fig. S5** Optical images of MXene-CNF ink printed on different polymer substrates using DIW technology. **a** PLA film, **b** PET film, **c** PI film. Scale bars, 10 mm


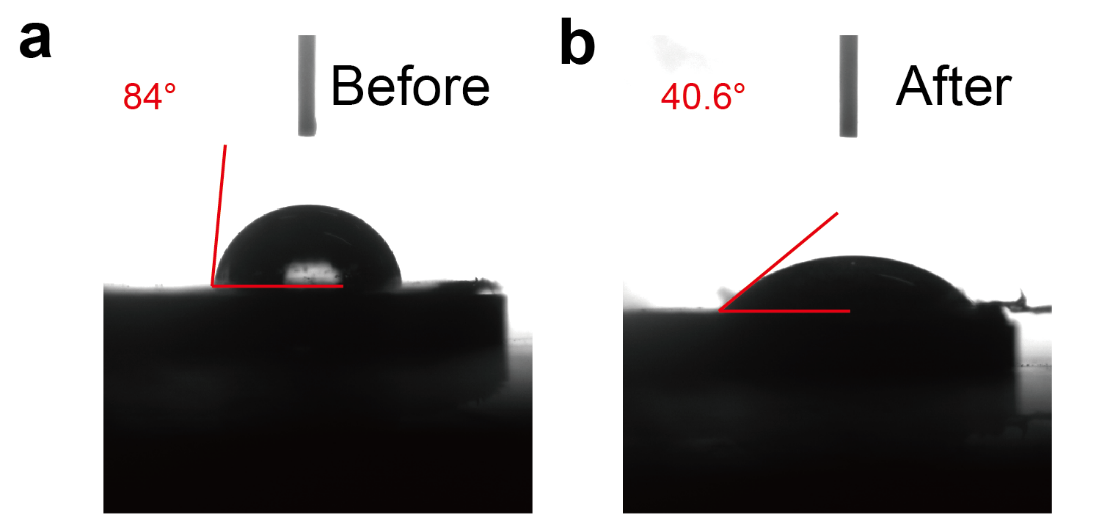


**Fig. S6** The water contact angle of PE film before and after plasma treatment


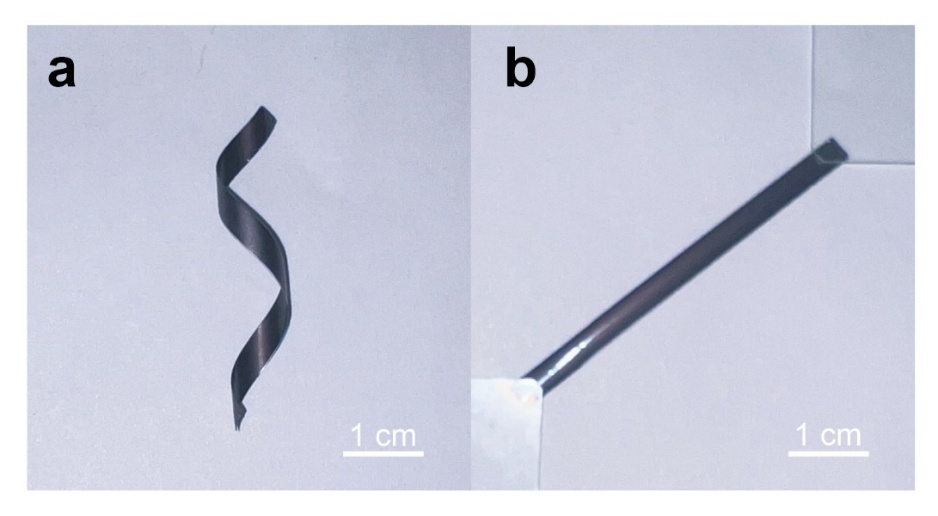


**Fig. S7** Optical images of the MXene-CNF/PE bilayer film. **a** coiling status, **b** uncoiling status


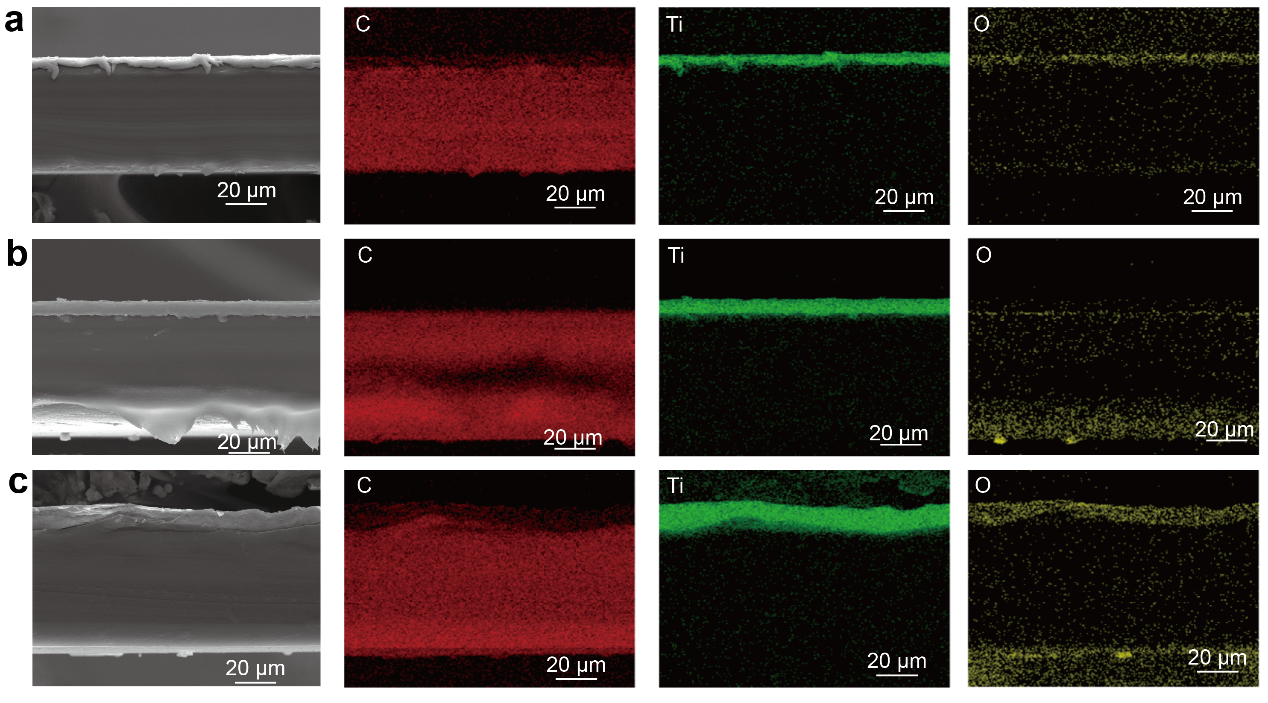


**Fig. S8** Cross-sectional SEM image of MXene-CNF/PE bilayer film. **a-c** Printing ply number of MXene-CNF film **(a)** One ply number, **(b)** Two ply numbers, and **(c)** Three ply numbers


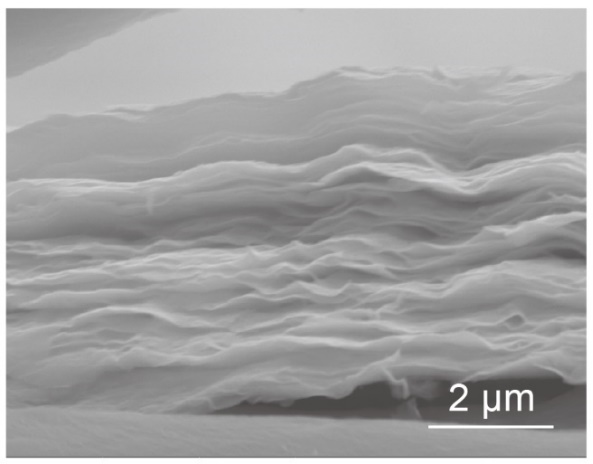


**Fig. S9** Cross-sectional SEM image of MXene-CNF layer


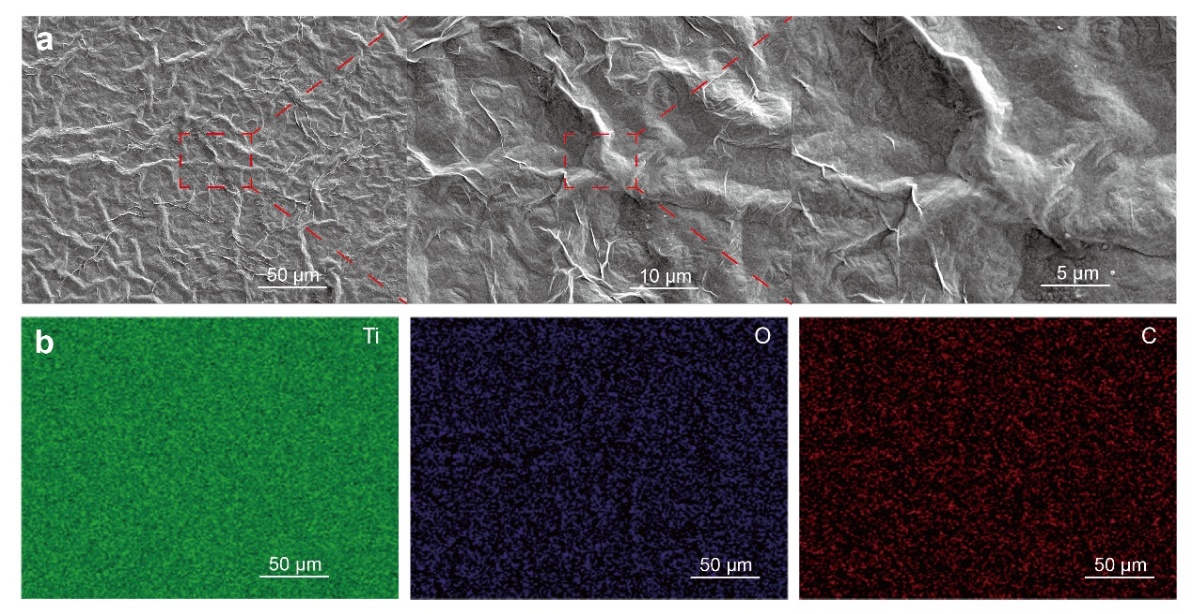


**Fig. S10** Morphology of the surface of MXene-CNF layer after drying. **a** SEM image, **b** EDS element mapping of Ti, O, and C


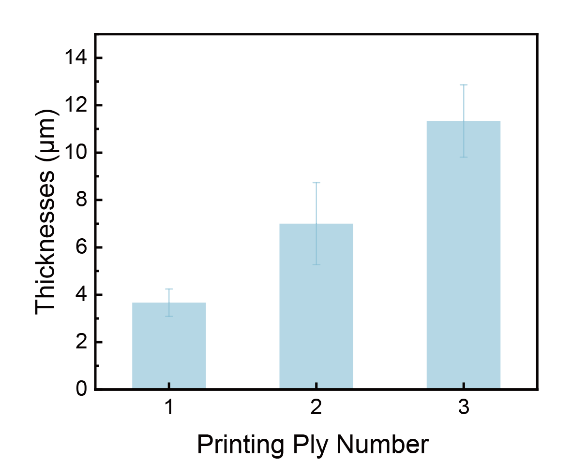


**Fig. S11** Dependence of MXene-CNF layer thickness on the printing ply number.


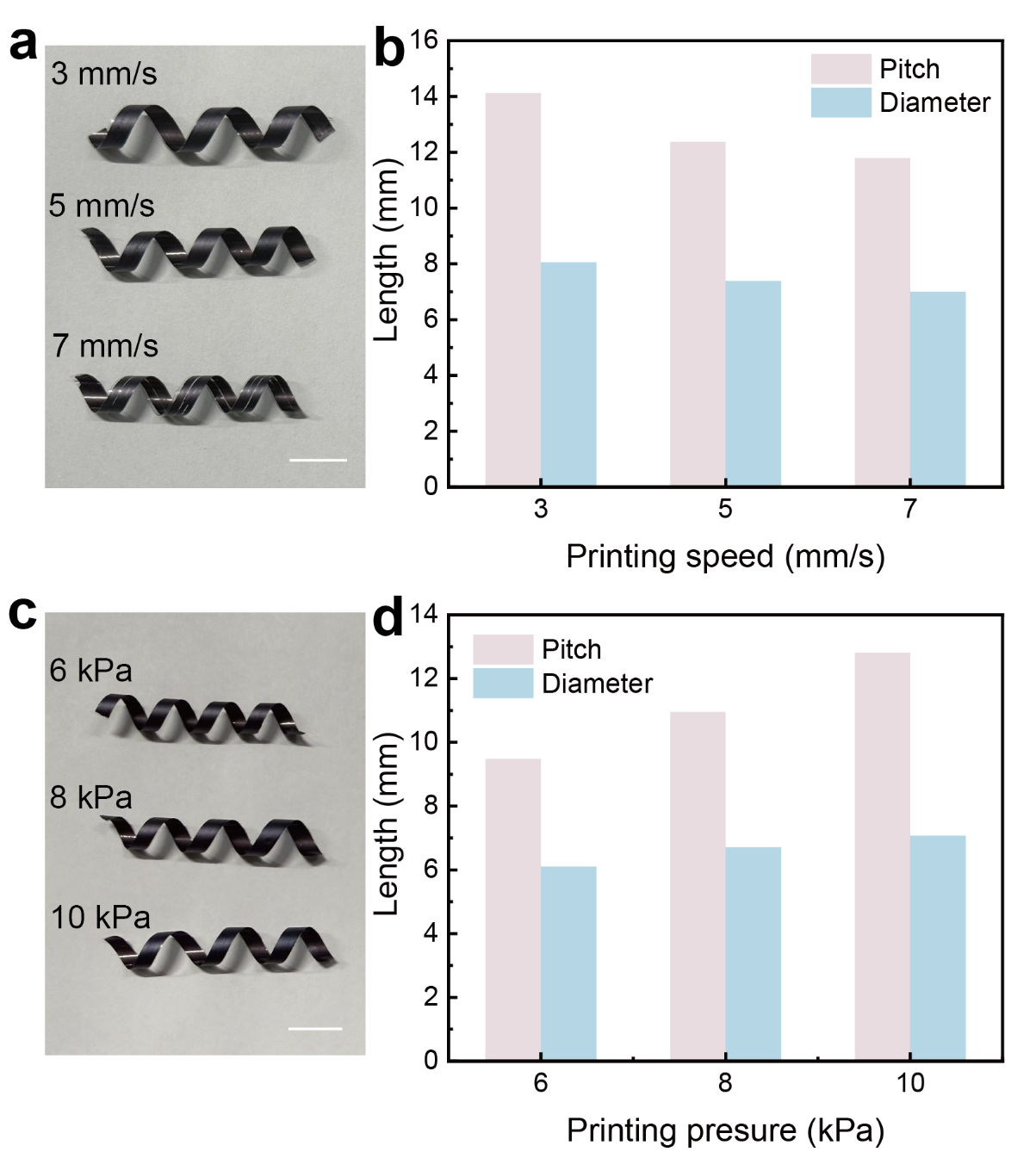


**Fig. S12** ICSBot fabricated with different printing speeds and pressures. **a, b** different printing speeds, **c, d** different pressures. Scale bars, 10 mm


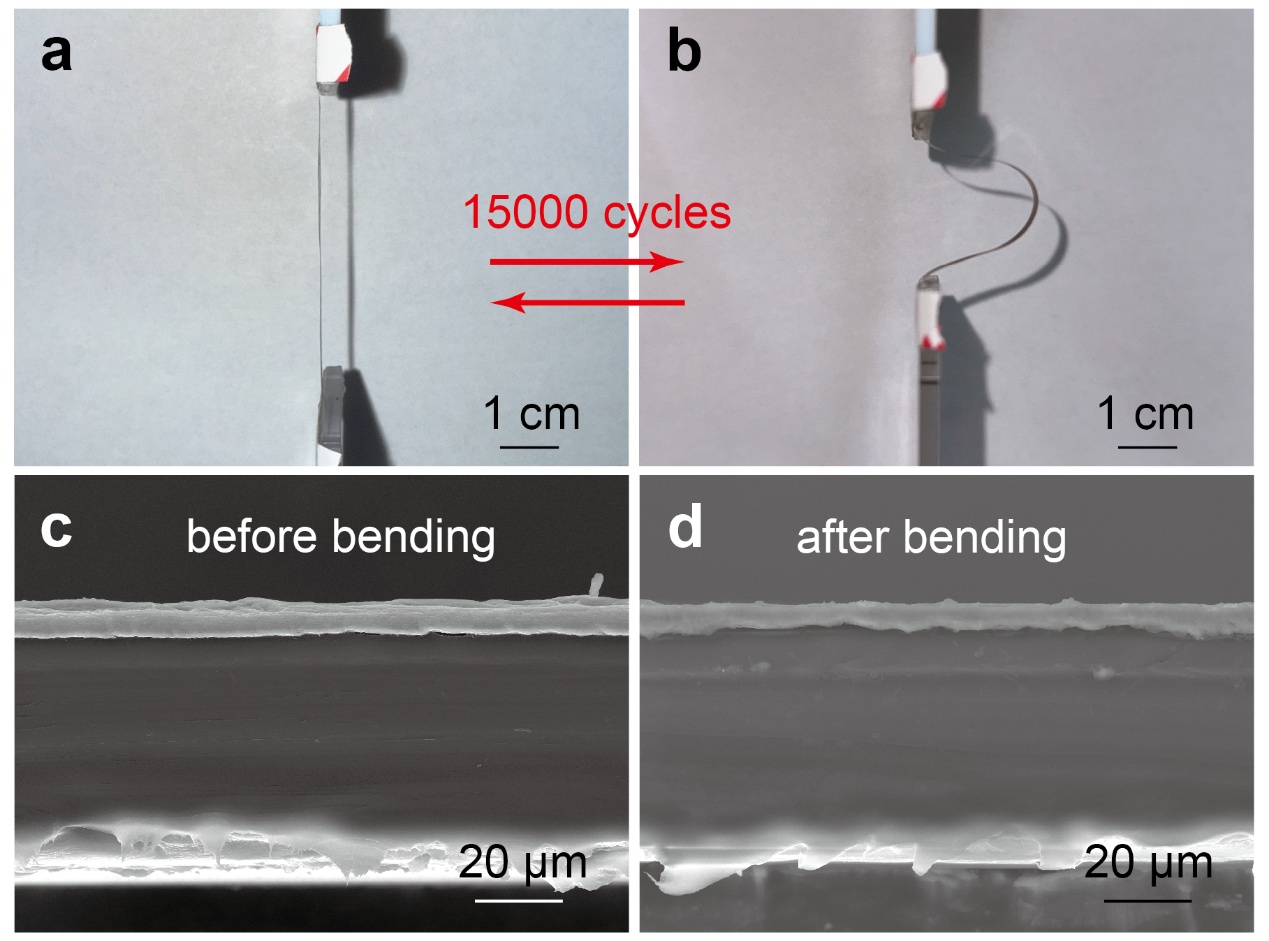


**Fig. S13 a, b** Optical image of the cyclic bending test of the MXene-CNF/PE bilayer film. **c, d** Cross-sectional SEM images of the MXene-CNF/PE bilayer film before (c) and after (d) 15000 times cyclic bending tests


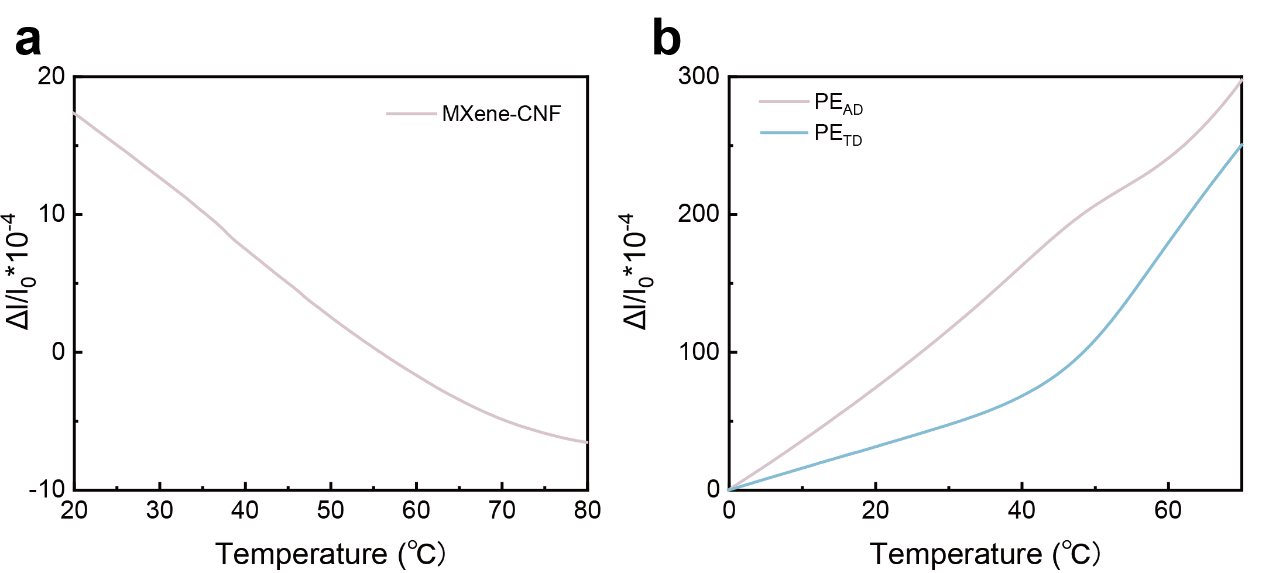


**Fig. S14** Thermal expansion/contraction behaviors of MXene-CNF layer and PE layer. **a** MXene-CNF layer, **b** AD and TD direction of PE layer


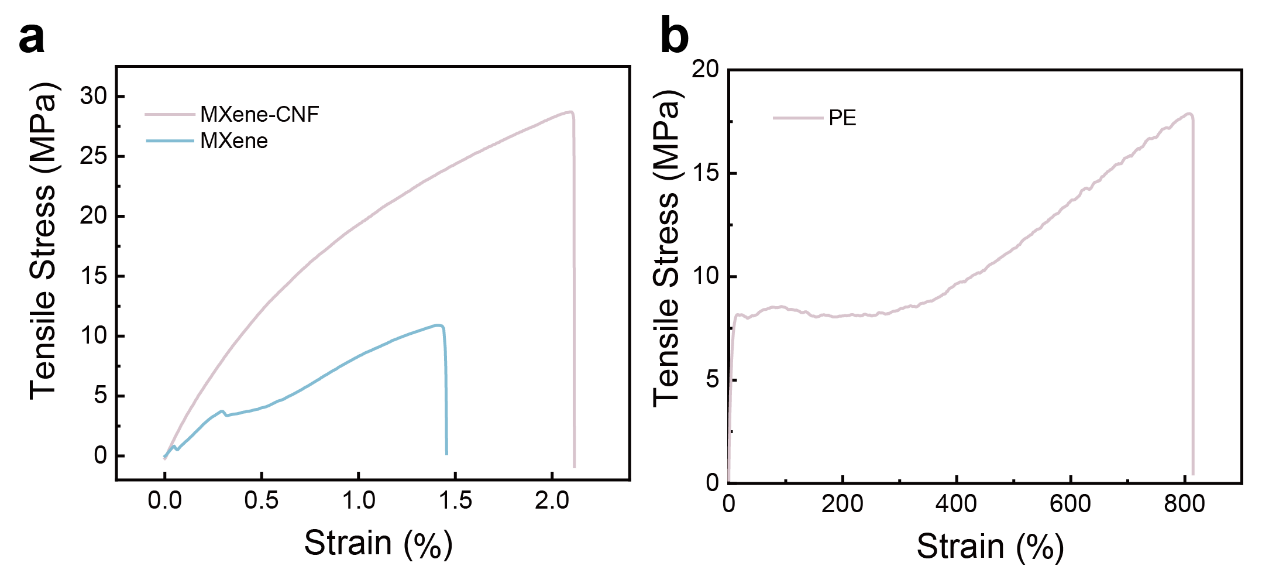


**Fig. S15** Mechanical properties of MXene, MXene-CNF, and PE**. a** Stress-strain curves of MXene and MXene-CNF, **b** Stress-strain curves of PE layer


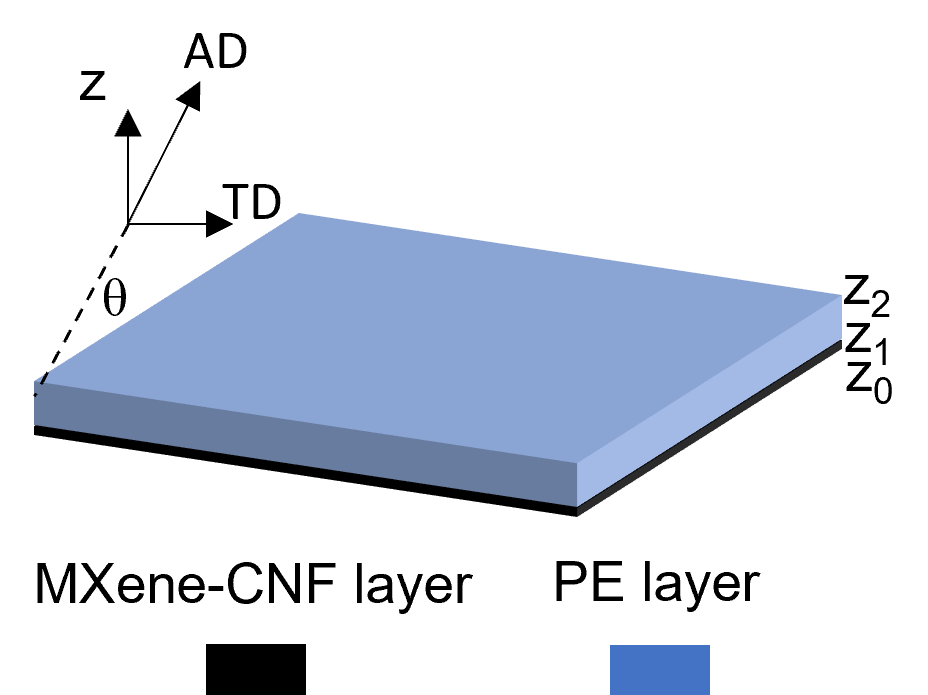


**Fig. S16** Schematics of the anisotropic composite plates


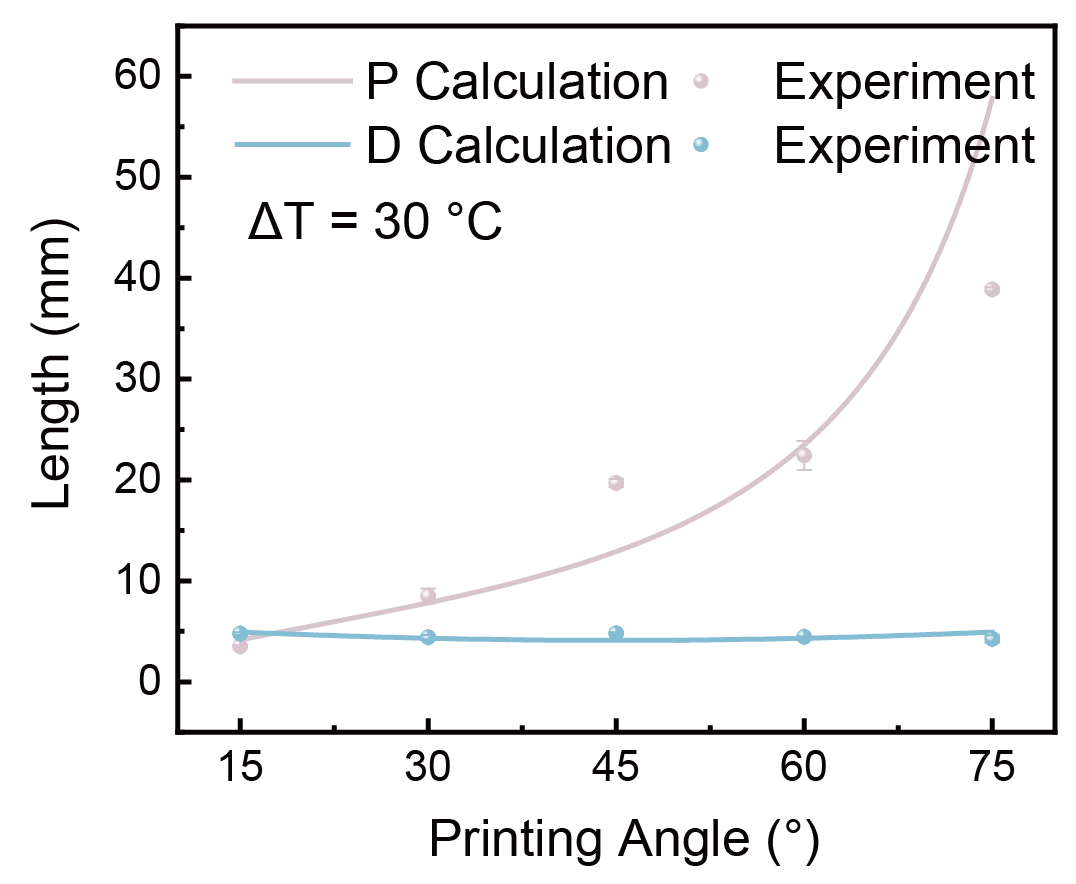


**Fig. S17** Dependence of pitch and diameter of ICSBot on printing angles (ΔT = 30 °C) under laminated composite material model


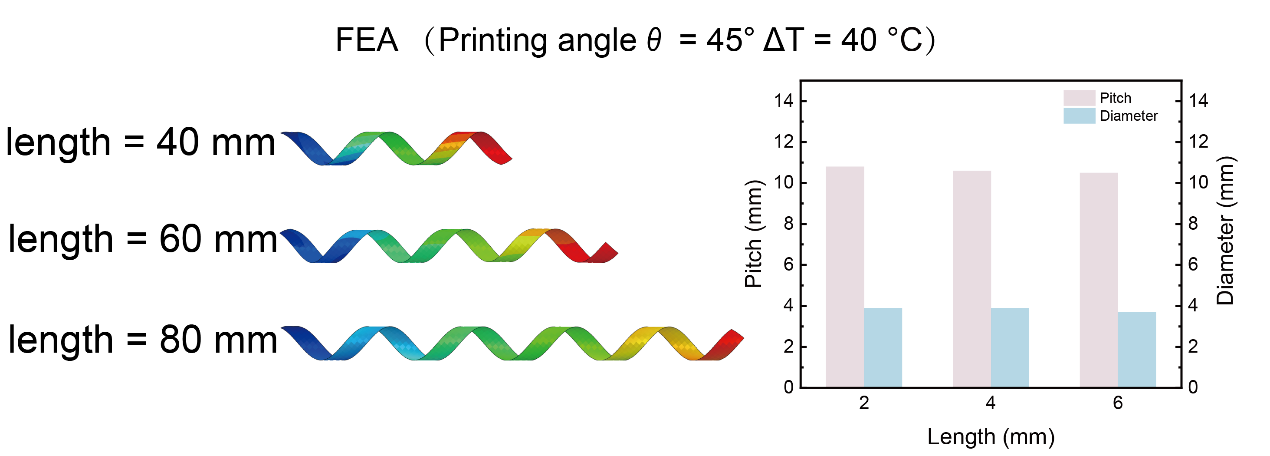


**Fig. S18** FEA result of ICSBot fabricated with different lengths of printed rectangular patterns


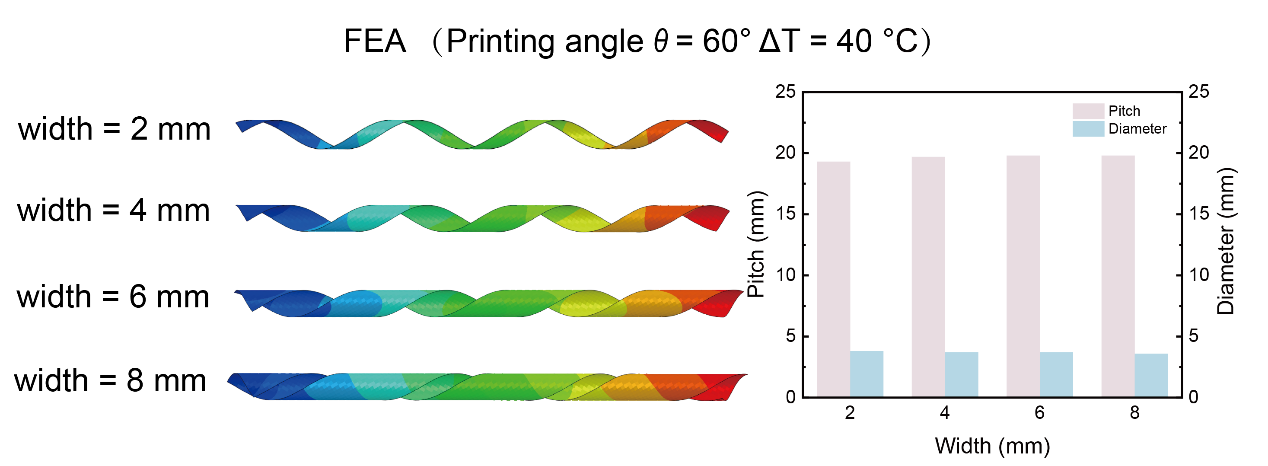


**Fig. S19** FEA result of ICSBot fabricated with different widths of printed rectangular patterns


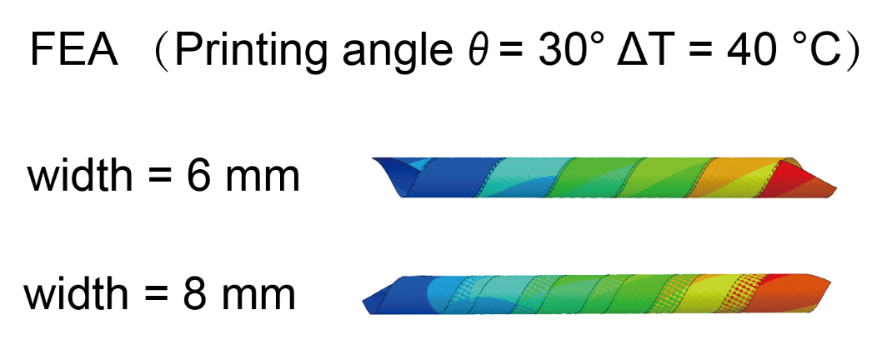


**Fig. S20** FEA result of ICSBot fabricated with rectangular pattern width D greater than pitch P


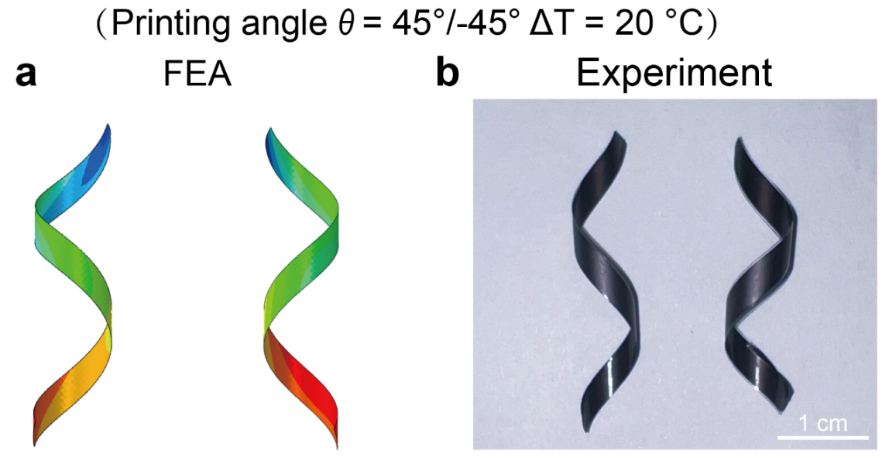


**Fig. S21** FEA and optical images of ICSBot at printing angles of 45° and -45°. **a** FEA, **b** optical image


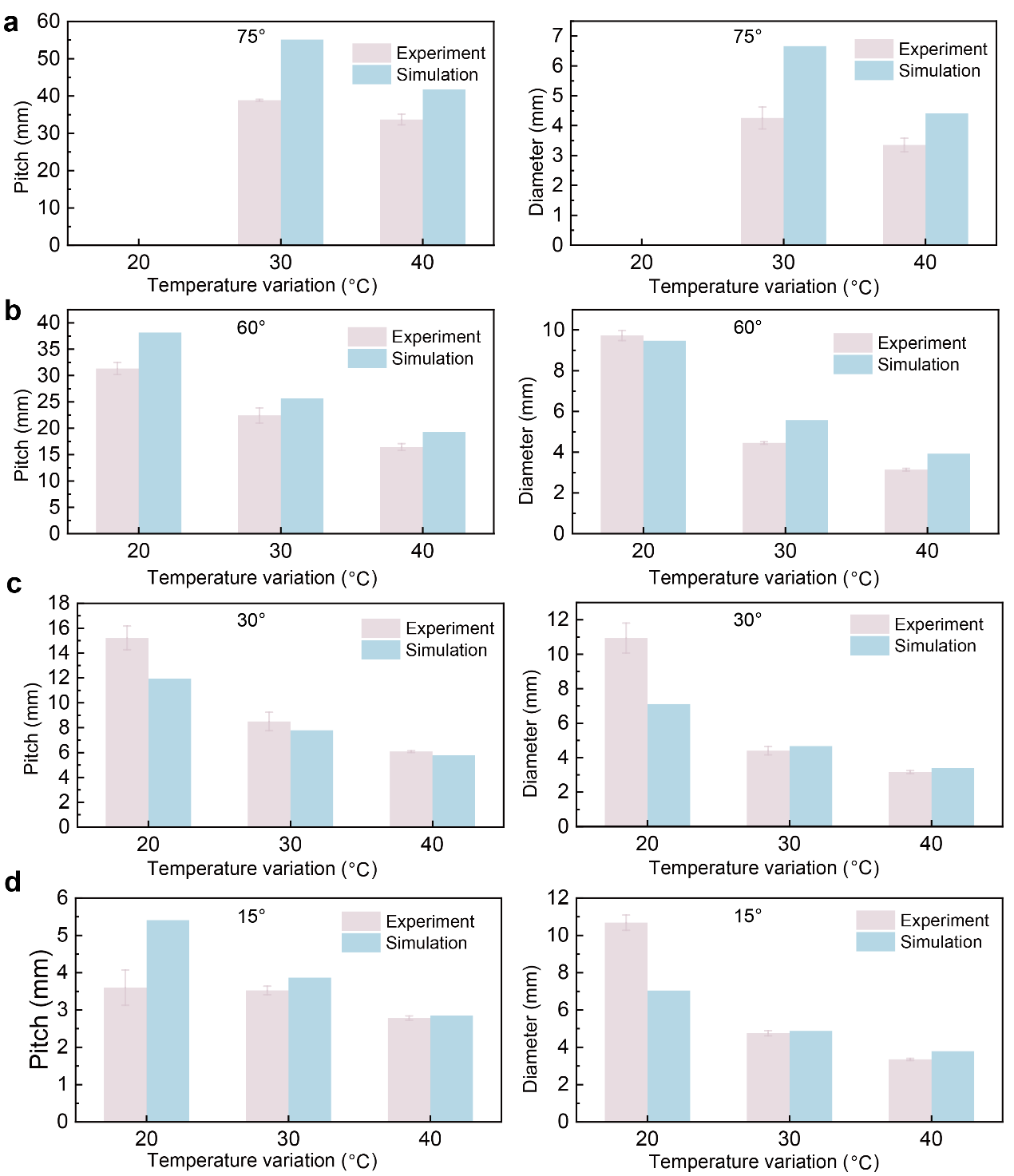


**Fig. S22** Comparison between FEA and experimental results in pitch and diameter of ICSBot as a function of temperature variation. **a** printing angle *θ* = 75°, **b** printing angle *θ* = 60°, **c** printing angle *θ* = 30°, **d** printing angle *θ* = 15°


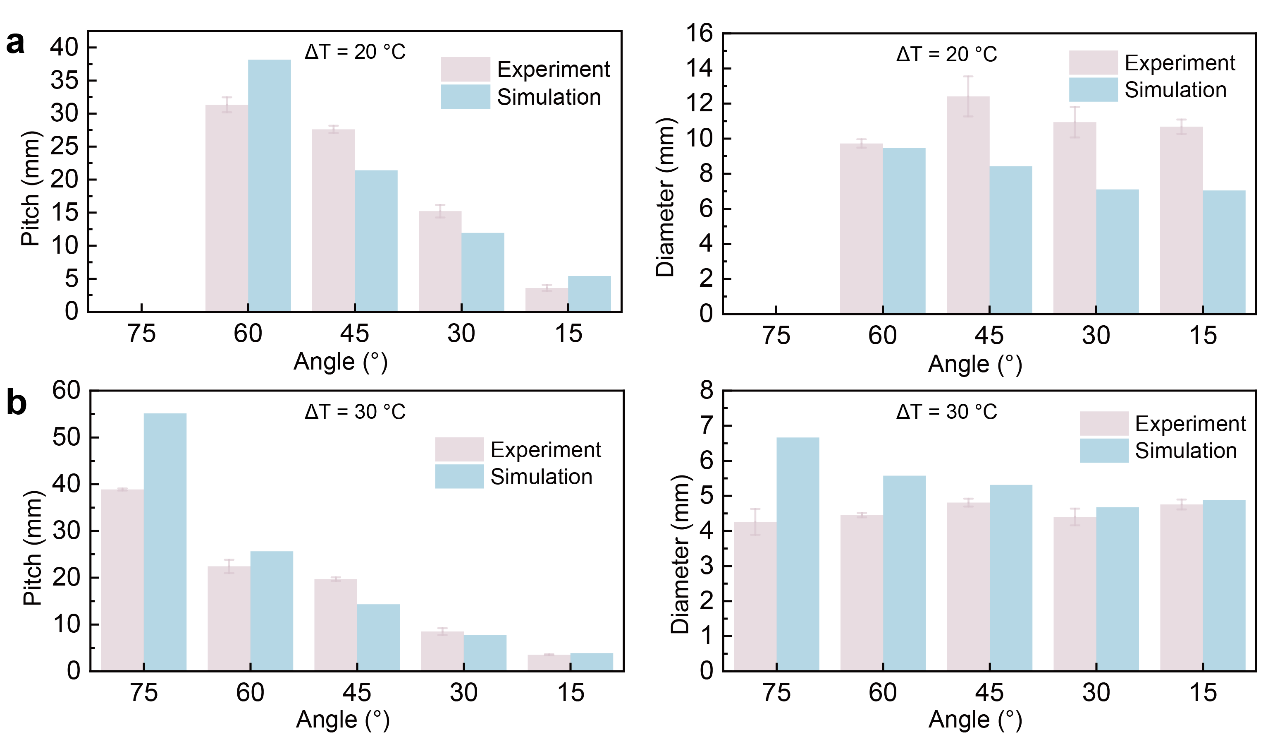


**Fig. S23** Comparison between FEA and experimental results in pitch and diameter of ICSBot as a function of printing angle. **a** temperature variation ΔT = 20 °C, **b** temperature variation ΔT = 30 °C


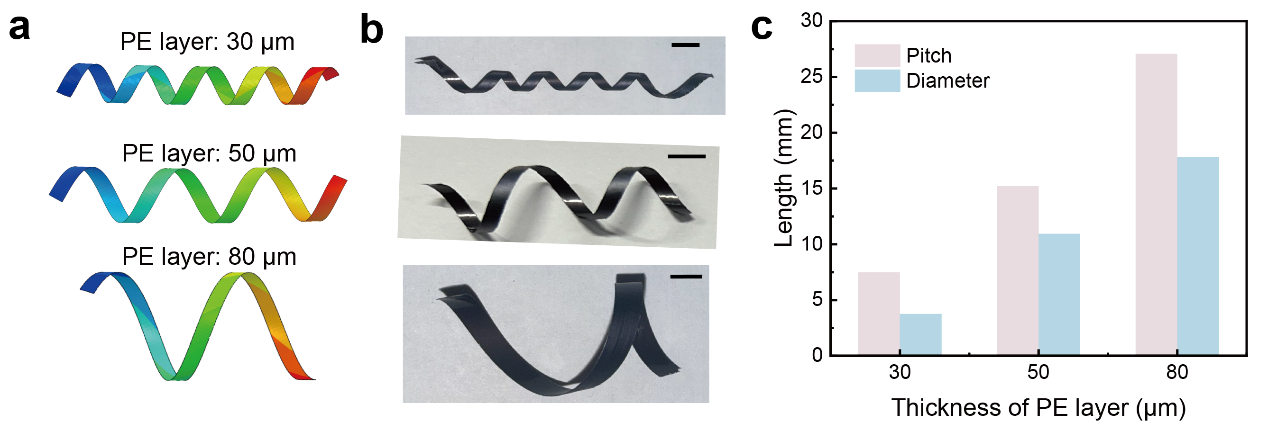


**Fig. S24** FEA and optical images of ICSBot fabricated with different thicknesses of PE layer. Scale bars, 5 mm


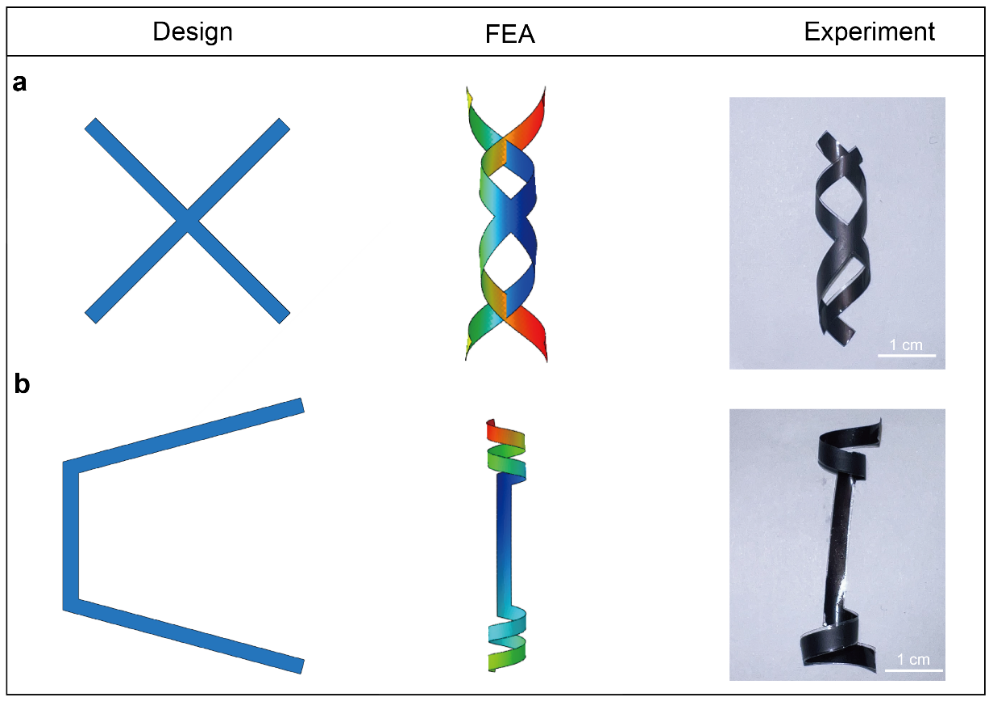


**Fig. S25** Design and fabrication of ICSBot with complex initial structure. **a** X shape, **b** U shape


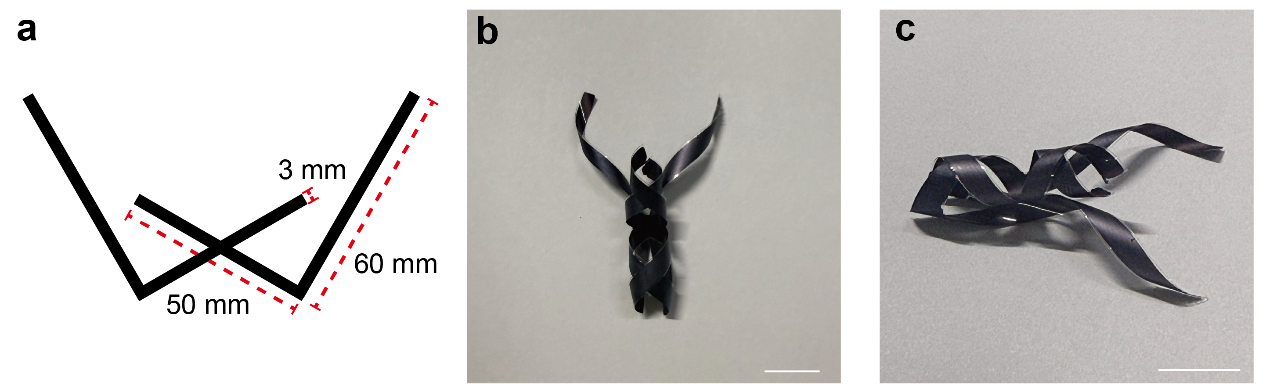


**Fig. S26** Programmable ICSBot with complex geometric structures. **a** complex geometric structure, **b, c** Optical image of “ant” structured ICSBot. Scale bars, 10 mm

**
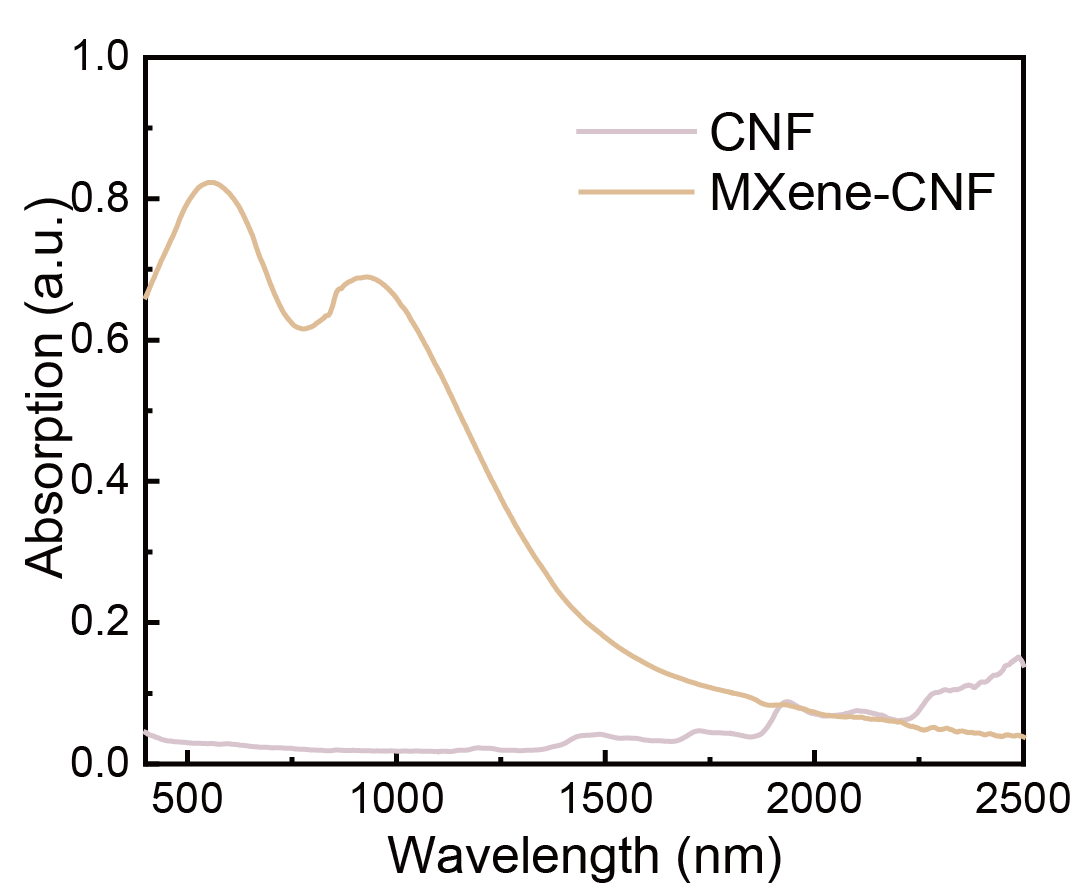
**

**Fig. S27** Ultraviolet-visible-near-infrared absorption spectra of the MXene-CNF layer and CNF


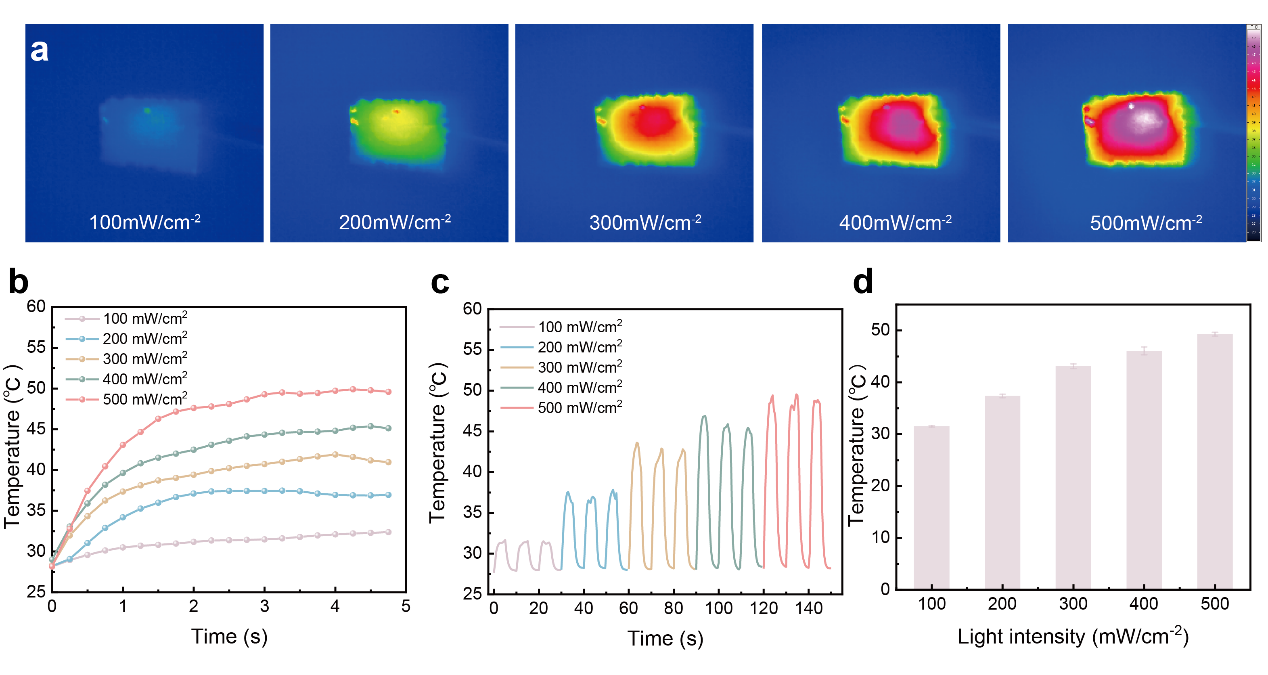


**Fig. S28** The photothermal conversion performance of MXene-CNF layer. **a** Infrared thermal image of MXene-CNF layer under different light intensity irradiation, **b** The equilibrium temperature of the MXene-CNF layer at various light intensities, **c** Temperature change of MXene-CNF layer under different light intensity irradiation on/off conversion cycles, **d** Maximum temperature under different light intensity irradiation


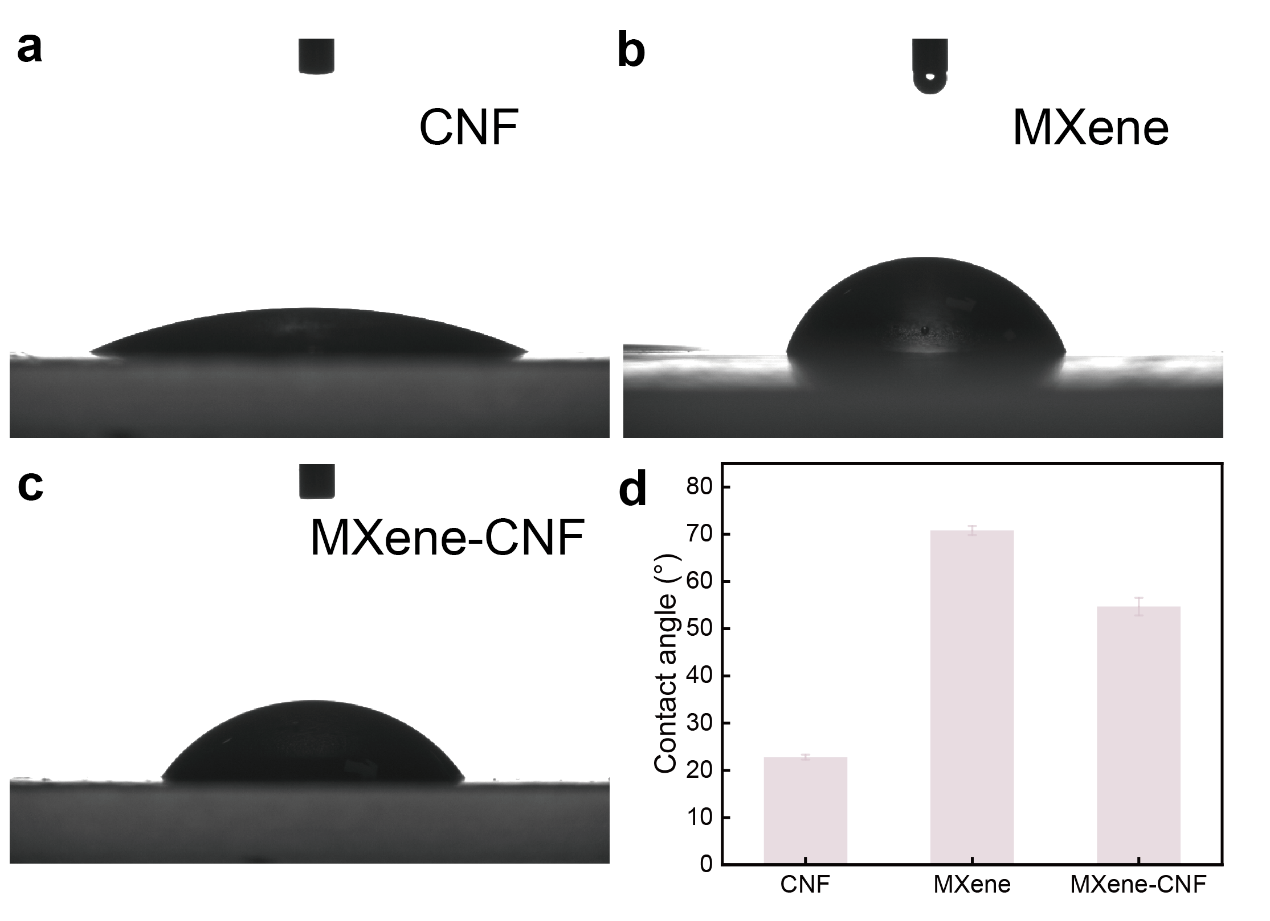


**Fig. S29** Water contact angle of CNF, MXene, and MXene-CNF


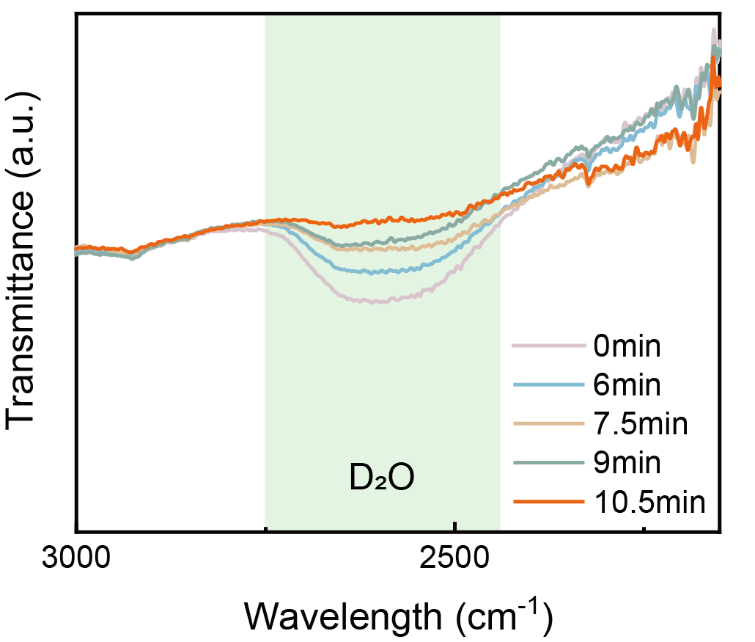


**Fig. S30** Time-dependent ATR-FTIR spectra for MXene-CNF film after exposure to D_2_O vapor for 1minute followed by rapid release of D_2_O in air.


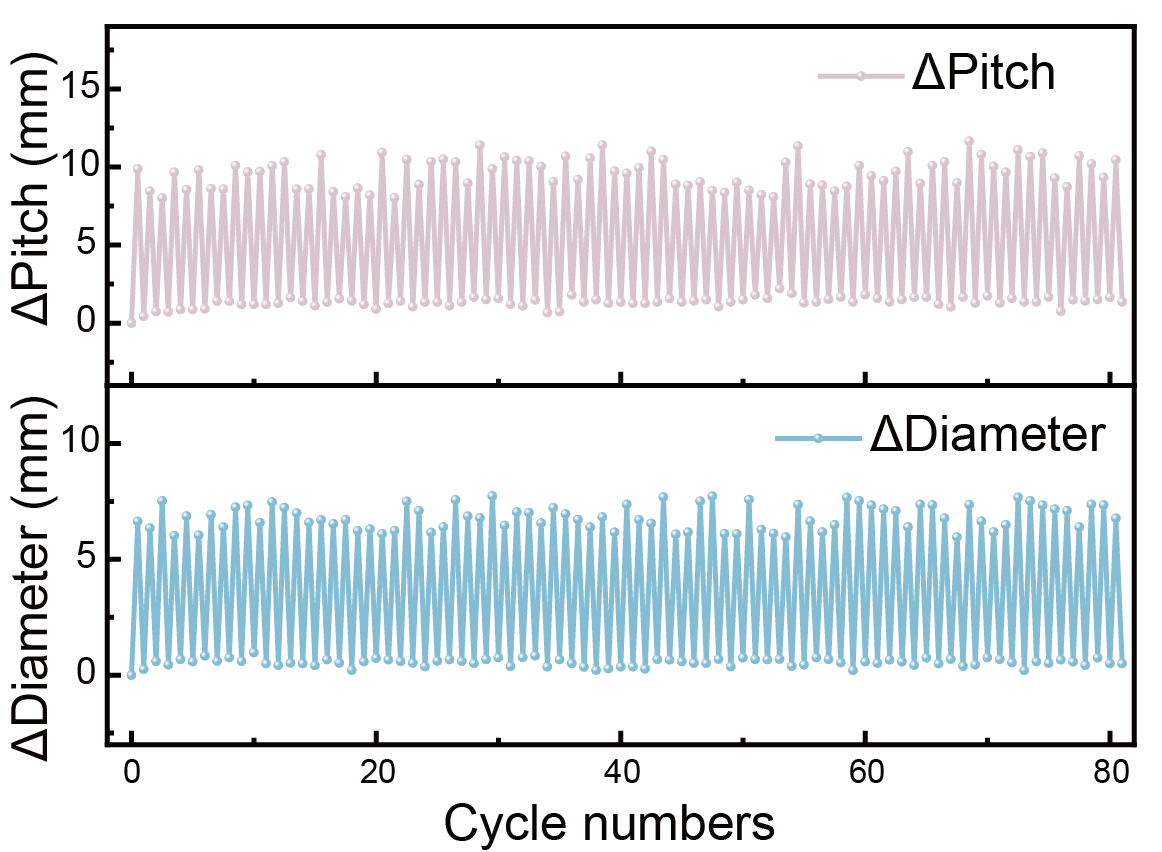


**Fig. S31** Cyclic test with repeated exposure to light irradiation (200 mW cm^-2^).


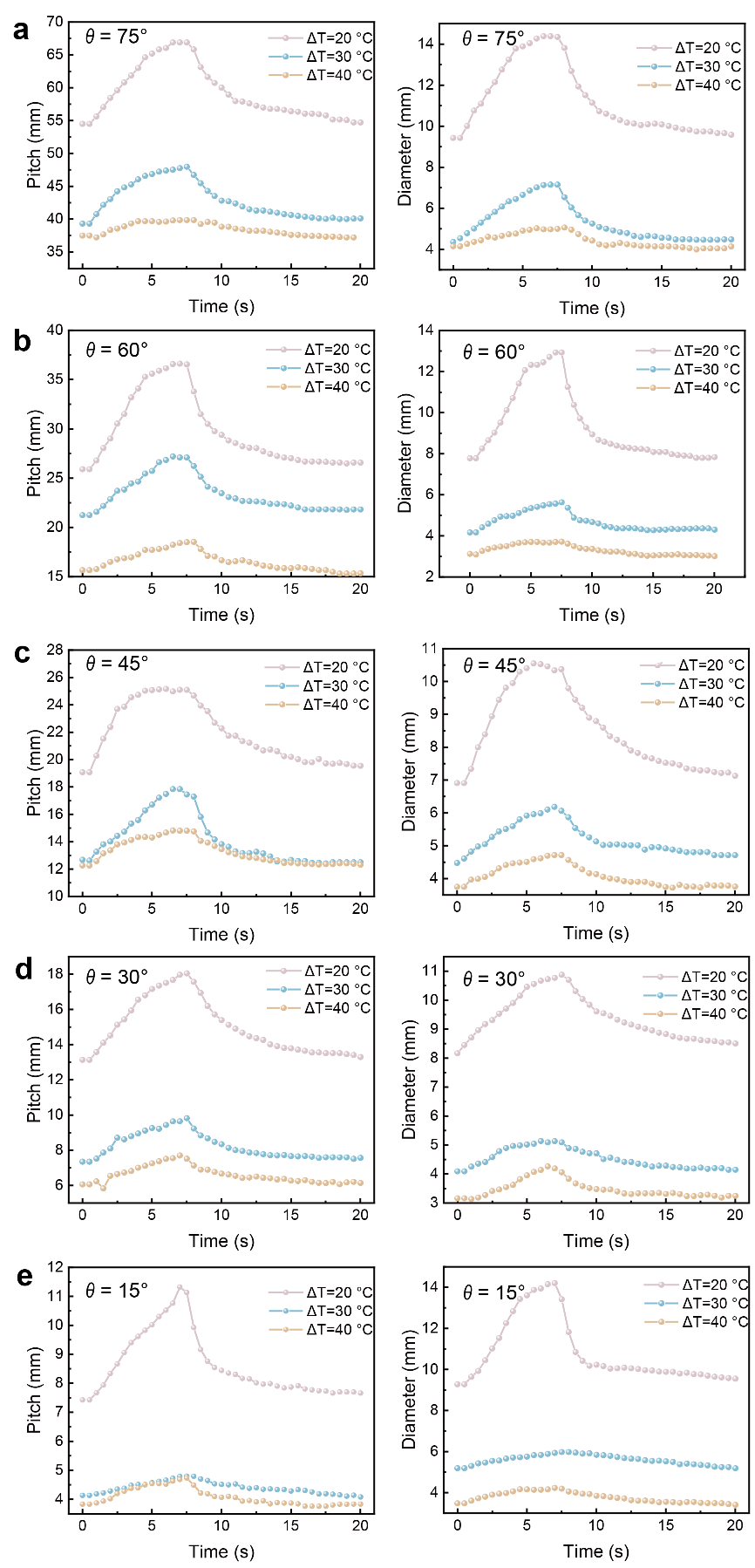


**Fig. S32** Real-time pitch and diameter of ICSBot with different printing angles and temperature variations under NIR light irradiation (100 mW cm^-2^). **a** printing angle *θ* = 75°, **b** printing angle *θ* = 60°, **c** printing angle *θ* = 45°, **d** printing angle *θ* = 30°, **e** printing angle *θ* = 15°


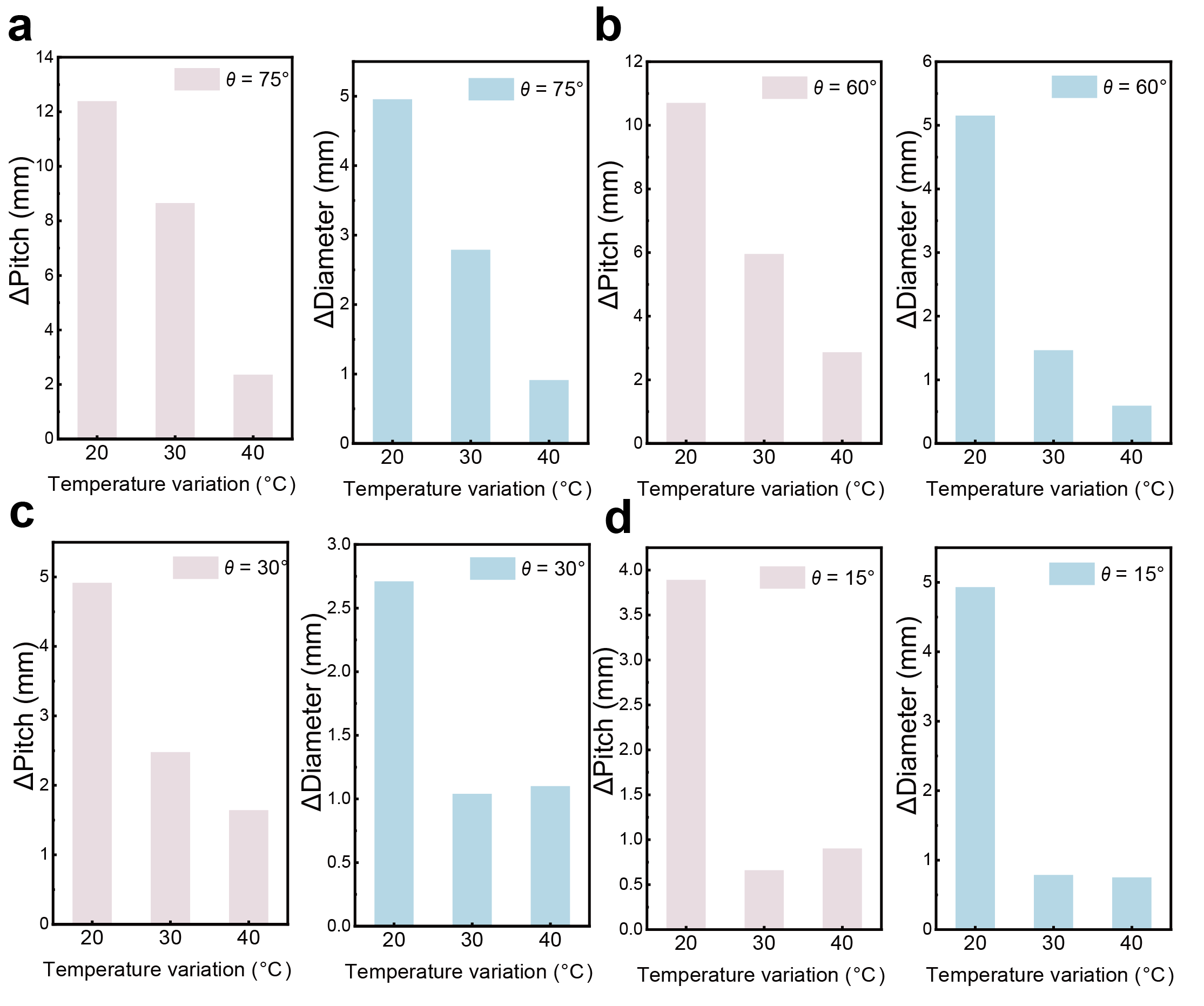


**Fig. S33** Dependence of changes in pitch and diameter of the ICSBot on temperature variation under NIR light irradiation (100 mW cm^-2^). **a** printing angle *θ* = 75°, **b** printing angle *θ* = 60°, **c** printing angle *θ* = 30°, **d** printing angle *θ* = 15°


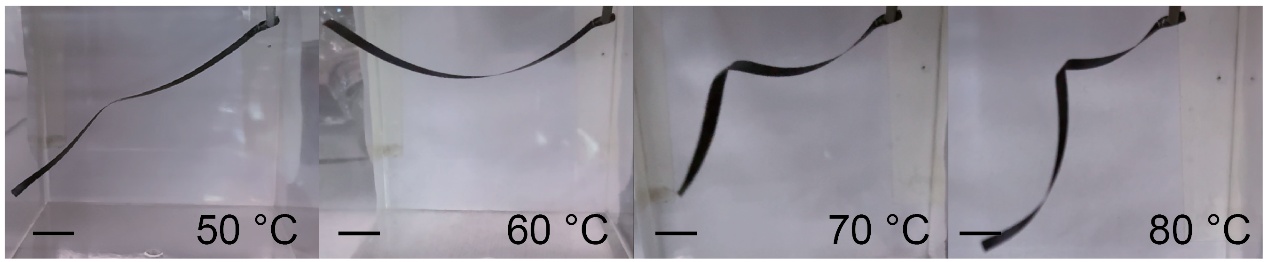


**Fig. S34** Optical images of the coiling deformation of the ICSBot under different temperatures (50 °C, 60 °C, 70 °C and 80 °C). Scale bars, 10 mm


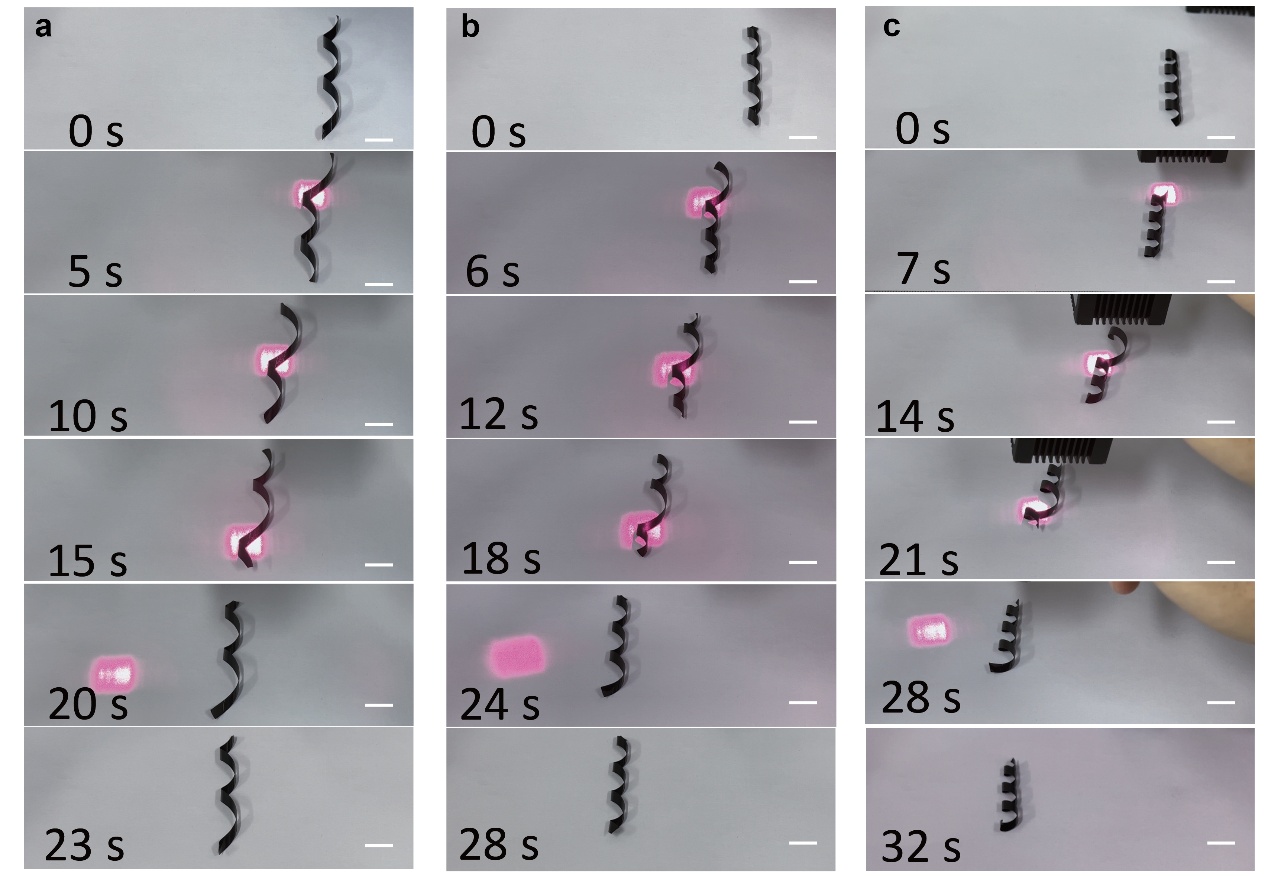


**Fig. S35** Optical images of sidewinding crawling of ICSBot with different printing angles. **a** printing angle *θ* = 60°, **b** printing angle *θ* = 45°, **c** printing angle *θ* = 30°. Scale bars, 10 mm


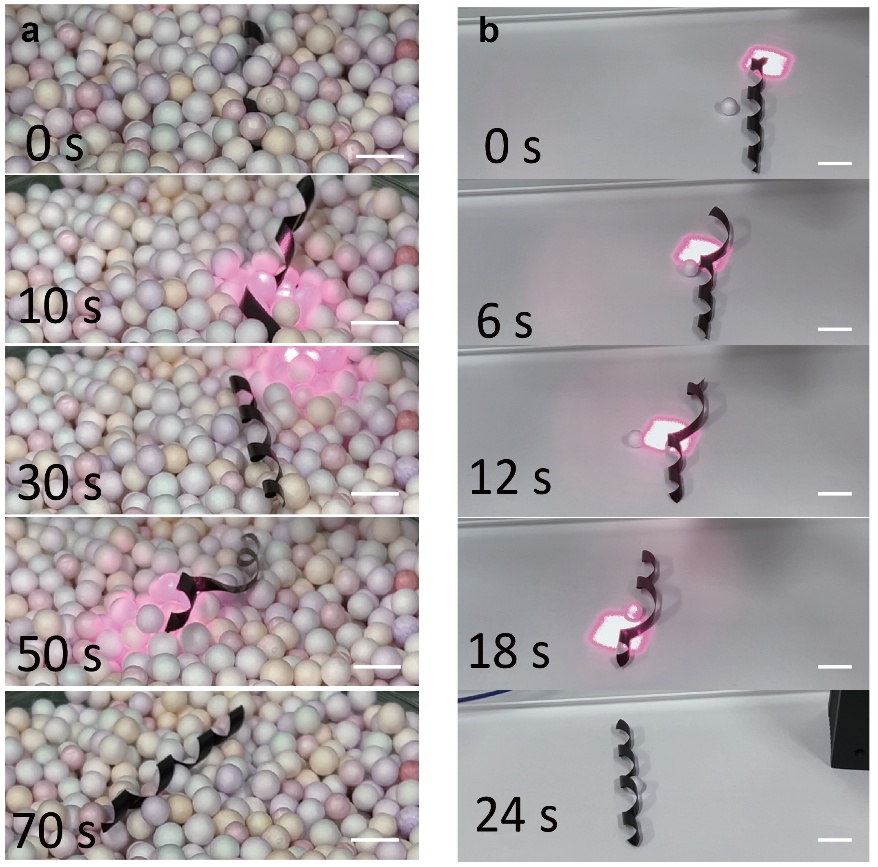


**Fig. S36** Application of ICSBot based on sidewinding crawling. **a** Escaping from complex environments, **b** Crawling under the condition of pushing objects. Scale bars, 10 mm


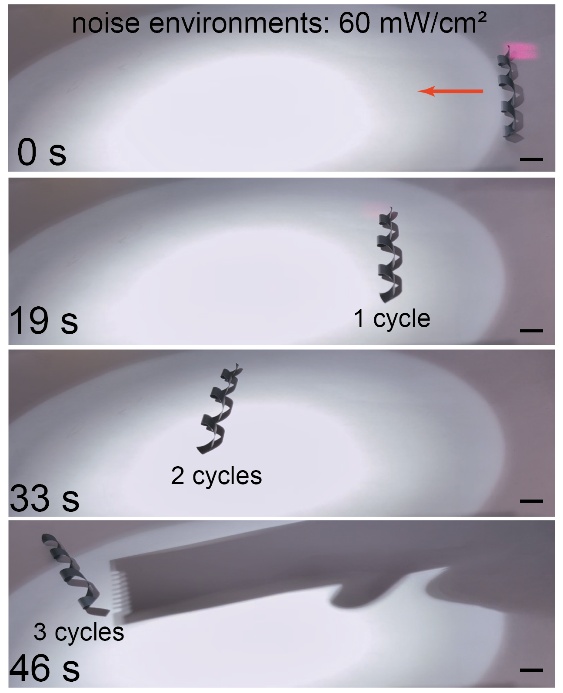


**Fig. S37** Optical images of sidewinding crawling of ICSBot in high-noise environments. Scale bars, 10 mm


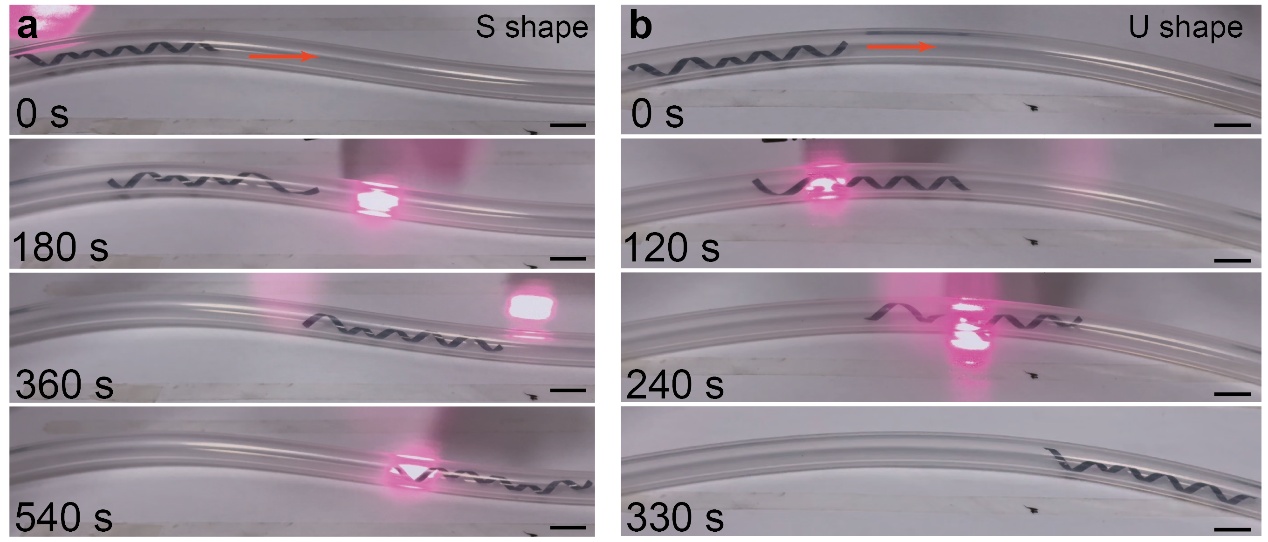


**Fig. S38** Optical images of accordion crawling of ICSBot inside the S shape and U shape rubber tube. Scale bars, 10 mm


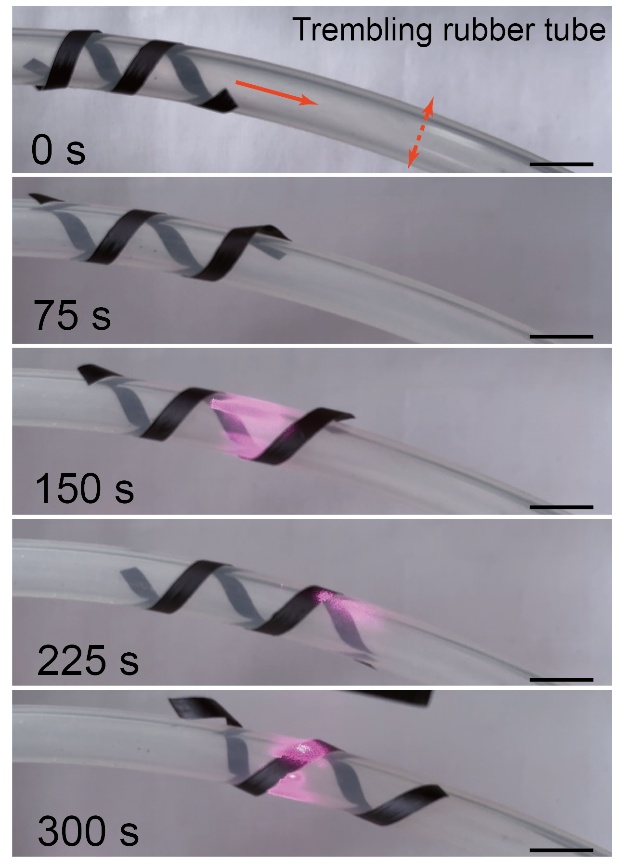


**Fig. S39** Optical images of winding climbing of ICSBot outside the trembling rubber tube. Scale bars, 10 mm

**Table S1** Properties of the MXene-CNF and PE layers

|  | MXene-CNF layer | PE layer | |
| --- | --- | --- | --- |
|  |  | AD | TD |
| Young’s Modulus (GPa) | 2.984 | 0.239 | |
| Poisson’s coefficient | 0.23[S5] | 0.46 [S6] | |
| Thickness (μm) | 10 | 30/50/80 | |
| CTE (ppm K^-1^) | -49 | 430 | 156 |

**Table S2** Comparison between ICSBot and other soft robots

| **Types** | **Materials** | **Structure** | **Stimulation** | **Locomotion mode** | **Refs.** |
| --- | --- | --- | --- | --- | --- |
| Snake-inspired soft robot | modular snake robot | self-repeating | Electric | Sidewinding | [S7] |
|  | polyester plastic /Ecoflex | kirigami | Pneumatic | Rectilinear | [S8] |
|  | elastic bellow | two winding actuators, a telescopic actuator | Pneumatic | Winding climbing | [S9] |
|  | LCE | Coiling | Heat | Sidewinding | [S10] |
|  | GO-PDA/rGO/GO-PDA | Triple-layer | NIR Light | Accordion | [S11] |
| Coiling structure soft robot | LCE | Coiling | Heat | Rolling | [S12] |
|  | LCN/PI | Coiling | Electric | Grasping, Crawling | [S13] |
|  | LCE/PP | Coiling | UV Light | Rolling | [S14] |
|  | GO/BOPP | Coiling | IR Light | Grasping | [S15] |
|  | Vitrimer-A/Vitrimer-B | Coiling | Light, Humidity | Grasping | [S16] |
| Multimodal locomotion soft robot | PDMS/PI | Coiling | Light, Magnetic | Flipping, Rolling, Rotating | [S17] |
|  | PDMS/ Metal scales | Bilayer | Magnetic | Rolling, Tumbling | [S18] |
|  | LCE/ PDMS | Bilayer | Light | Crawling, Turning, Rotating | [S19] |
|  | LCE/ MXene | Bilayer | NIR Light | Crawling, Jumping | [S20] |
| This work | MXene-CNF/PE | Coiling | NIR Light, Humidity, Heat | Grasping, Sidewinding, Accordion, Winding | This work |

**Supplementary References**

1. J. Xiong, X. Zhao, Z. Liu, H. Chen, Q. Yan et al., Multifunctional nacre-like nanocomposite papers for electromagnetic interference shielding *via* heterocyclic aramid/MXene template-assisted *in situ* polypyrrole assembly. Nano-Micro Lett. **17**(1), 53 (2024). <https://doi.org/10.1007/s40820-024-01552-9>
2. Z. Chen, X. Zhao, B. Gao, L. Xu, H. Chen et al., Biobased inks based on cuttlefish ink and cellulose nanofibers for biodegradable patterned soft actuators. ACS Appl. Mater. Interfaces **16**(17), 22547–22557 (2024). <https://doi.org/10.1021/acsami.4c02775>
3. J. Ha, S.M. Choi, B. Shin, M. Lee, W. Jung et al., Hygroresponsive coiling of seed awns and soft actuators. Extreme Mech. Lett. **38**, 100746 (2020). <https://doi.org/10.1016/j.eml.2020.100746>
4. L. Cecchini, S. Mariani, M. Ronzan, A. Mondini, N.M. Pugno et al., 4D printing of humidity-driven seed inspired soft robots. Adv. Sci. **10**(9), 2205146 (2023). <https://doi.org/10.1002/advs.202205146>
5. L. Xu, H. Zheng, F. Xue, Q. Ji, C. Qiu et al., Bioinspired multi-stimulus responsive MXene-based soft actuator with self-sensing function and various biomimetic locomotion. Chem. Eng. J. **463**, 142392 (2023). <https://doi.org/10.1016/j.cej.2023.142392>
6. S. Wang, Y. Gao, A. Wei, P. Xiao, Y. Liang et al., Asymmetric elastoplasticity of stacked graphene assembly actualizes programmable untethered soft robotics. Nat. Commun. **11**(1), 4359 (2020). <https://doi.org/10.1038/s41467-020-18214-0>
7. H. Marvi, C. Gong, N. Gravish, H. Astley, M. Travers et al., Sidewinding with minimal slip: snake and robot ascent of sandy slopes. Science **346**(6206), 224–229 (2014). <https://doi.org/10.1126/science.1255718>
8. A. Rafsanjani, Y. Zhang, B. Liu, S.M. Rubinstein, K. Bertoldi, Kirigami skins make a simple soft actuator crawl. Sci. Robot. **3**(15), eaar7555 (2018). <https://doi.org/10.1126/scirobotics.aar7555>
9. B. Liao, H. Zang, M. Chen, Y. Wang, X. Lang et al., Soft rod-climbing robot inspired by winding locomotion of snake. Soft Robot. **7**(4), 500–511 (2020). <https://doi.org/10.1089/soro.2019.0070>
10. Y.B. Kim, S. Yang, D.S. Kim, Sidewinder-inspired self-adjusting, lateral-rolling soft robots for autonomous terrain exploration. Adv. Sci. **11**(14), 2308350 (2024). <https://doi.org/10.1002/advs.202308350>
11. Y. Yang, M. Zhang, D. Li, Y. Shen, Graphene-based light-driven soft robot with snake-inspired *Concertina* and serpentine locomotion. Adv. Mater. Technol. **4**(1), 1800366 (2019). <https://doi.org/10.1002/admt.201800366>
12. Y. Zhao, Y. Chi, Y. Hong, Y. Li, S. Yang et al., Twisting for soft intelligent autonomous robot in unstructured environments. Proc. Natl. Acad. Sci. USA **119**(22), e2200265119 (2022). <https://doi.org/10.1073/pnas.2200265119>
13. Y.-Y. Xiao, Z.-C. Jiang, X. Tong, Y. Zhao, Biomimetic locomotion of electrically powered “Janus” soft robots using a liquid crystal polymer. Adv. Mater. **31**(36), 1903452 (2019). <https://doi.org/10.1002/adma.201903452>
14. X. Lu, S. Guo, X. Tong, H. Xia, Y. Zhao, Tunable photocontrolled motions using stored strain energy in malleable azobenzene liquid crystalline polymer actuators. Adv. Mater. **29**(28), 1606467 (2017). <https://doi.org/10.1002/adma.201606467>
15. Q. Li, Y. Jiao, Ultrafast photothermal actuators with a large helical curvature based on ultrathin GO and biaxially oriented PE films. ACS Appl. Mater. Interfaces **14**(50), 55828–55838 (2022). <https://doi.org/10.1021/acsami.2c18478>
16. Q. Chen, X. Qian, Y. Xu, Y. Yang, Y. Wei et al., Harnessing the day-night rhythm of humidity and sunlight into mechanical work using recyclable and reprogrammable soft actuators. ACS Appl. Mater. Interfaces **11**(32), 29290–29297 (2019). <https://doi.org/10.1021/acsami.9b09324>
17. Z. Tian, J. Xue, X. Xiao, C. Du, Y. Liu, Optomagnetic coordination helical robot with shape transformation and multimodal motion capabilities. Nano Lett. **24**(9), 2885–2893 (2024). <https://doi.org/10.1021/acs.nanolett.4c00047>
18. R.H. Soon, Z. Yin, M.A. Dogan, N.O. Dogan, M.E. Tiryaki et al., Pangolin-inspired untethered magnetic robot for on-demand biomedical heating applications. Nat. Commun. **14**(1), 3320 (2023). <https://doi.org/10.1038/s41467-023-38689-x>
19. N. Qian, H.K. Bisoyi, M. Wang, S. Huang, Z. Liu et al., A visible and near-infrared light-fueled omnidirectional twist-bend crawling robot. Adv. Funct. Mater. **33**(16), 2214205 (2023). <https://doi.org/10.1002/adfm.202214205>
20. W. Cho, D.J. Kang, M.J. Hahm, J. Jeon, D.-G. Kim et al., Multi-functional locomotion of collectively assembled shape-reconfigurable electronics. Nano Energy **118**, 108953 (2023). <https://doi.org/10.1016/j.nanoen.2023.108953>
